# Are components of the histone gene expression machinery functionally repurposed in terminally differentiated cells?

**DOI:** 10.1242/bio.062755

**Published:** 2026-08-14

**Authors:** Xiao-Cui Yang, Anthony Desotell, Agata Malinowska, Richa Basundra, Gautam K. Pandey, Karen L. Mohlke, Mohanish Deshmukh, Michal Dadlez, Liang Tong, Zbigniew Dominski

**Affiliations:** ^1^ Integrative Program for Biological and Genome Sciences, University of North Carolina at Chapel Hill, Chapel Hill, NC 27599, USA; ^2^ Department of Biological Sciences, Columbia University, New York, NY 10027, USA; ^3^ Department of Biophysics, Institute of Biochemistry and Biophysics, Polish Academy of Sciences, 02-106 Warsaw, Poland; ^4^ Neuroscience Center, University of North Carolina at Chapel Hill, Chapel Hill, NC 27599, USA; ^5^ Department of Genetics, University of North Carolina at Chapel Hill, Chapel Hill, NC 27599, USA; ^6^ Institute of Genetics and Biotechnology, Warsaw University, 02-106 Warsaw, Poland; ^7^ Department of Biochemistry and Biophysics, University of North Carolina at Chapel Hill, Chapel Hill, NC 27599, USA

**Keywords:** NPAT, FLASH, U7 snRNP, Lsm11, Histone locus bodies, SMN

## Abstract

The expression of metazoan replication-dependent histone genes is controlled by the nuclear protein at the ataxia-telangiectasia locus (NPAT) and U7 small nuclear ribonucleoprotein particle (snRNP). NPAT activates transcription of histone genes during S-phase, whereas U7 snRNP is a multi-subunit endonuclease that cleaves the resultant transcripts at the 3′ end, yielding mature histone mRNAs. In cycling cells, NPAT and U7 snRNP with its four unique components, U7 snRNA, Lsm10, Lsm11 and FLASH, are highly enriched in histone locus bodies (HLBs), the nuclear condensates formed near histone gene loci. Here, we show that in muscle and neural cells that have ceased to replicate their chromatin and permanently exited the cell cycle, HLBs are dismantled and NPAT, FLASH and Lsm11 are detected in the cytoplasm. This observation suggests that in postmitotic cells, NPAT and U7 snRNP become repurposed for functions unrelated to generating histone mRNAs. We identified a highly conserved region in Lsm11 that engages in various protein–protein interactions and likely acts as a universal platform that controls the assembly, localization and function of Lsm11 complexes, including U7 snRNP, during cell growth and differentiation. Since the assembly of U7 snRNP requires survival motor neuron, the protein mutated in spinal muscular atrophy, our results may provide a new perspective on the pathophysiology of this neuromuscular disorder.

## Teaser

**Summary:** In post-mitotic muscle and neural cells, components of the histone gene expression machinery are found in the cytoplasm rather than in the nucleus. They may acquire non-canonical functions there, potentially providing new clues on the molecular defect in SMA.

## INTRODUCTION

Metazoan replication-dependent histone genes are unique among protein-encoding genes (Dominski and Marzluff, 1999; Schumperli and Pillai, 2004). Their transcription is initiated at the G1/S-phase transition and coincides with the hyperphosphorylation of the nuclear protein at the ataxia-telangiectasia locus (NPAT), a master transcriptional regulator of all replication-dependent histone genes (Ma et al., 2000; Zhao et al., 1998, 2000). The resultant histone mRNA precursors (pre-mRNAs) lack introns and are co-transcriptionally cleaved at the 3′ end by the U7 small nuclear ribonucleoprotein particle (snRNP), a multi-subunit endonuclease, yielding mature histone mRNAs terminated with a highly conserved stem-loop structure rather than a poly(A) tail. In the cytoplasm, these unique mRNAs are translated into histone proteins that are subsequently imported to the nucleus to form chromatin with the newly synthesized DNA.

The U7 snRNP consists of a ∼60-nucleotide U7 snRNA and a group of both common and unique protein subunits (Dominski and Tong, 2021; Schumperli and Pillai, 2004). The 5′ end region of U7 snRNA base-pairs with a purine-rich region located downstream of the cleavage site in histone pre-mRNAs, hence recruiting correct substrates to U7 snRNP (Bond et al., 1991; Dominski and Marzluff, 1999, 2007; Schaufele et al., 1986). The central region of U7 snRNA, referred to as the Sm binding site, promotes the assembly of a heptameric ring consisting of Lsm10, Lsm11, SmE, SmF, SmG, SmB and SmD3 (Khusial et al., 2005; Schumperli and Pillai, 2004). This ring differs from the spliceosomal-type Sm ring, which contains Sm proteins D1 and D2 instead of Lsm10 and Lsm11. The assembly of both Sm ring types occurs in the cytoplasm via a poorly understood mechanism that depends on two multi-subunit assemblies: the PRMT5 methylosome and the survival motor neuron (SMN) complex (Battle et al., 2006; Gruss et al., 2017; Neuenkirchen et al., 2008; Pillai et al., 2003; Tisdale et al., 2013, 2022). Mutations in the SMN protein, the key subunit of the SMN complex, cause spinal muscular atrophy (SMA), a genetic disorder characterized by selective death of motor neurons and muscle degeneration (Burghes and Beattie, 2009; Tisdale and Pellizzoni, 2015). Despite decades of research, it remains unclear whether SMA results from a defective Sm ring assembly or is linked to a different, tissue-specific function of SMN (Beattie and Kolb, 2018; Matera, 2025; Monani, 2005; Simcikova et al., 2023).

U7 snRNA surrounded by the heptameric ring (together referred to as the U7 snRNP core) is imported from the cytoplasm to the nucleus where it transits through Cajal bodies (CBs) and ultimately accumulates in histone locus bodies (HLBs) (Matera et al., 2007), membraneless structures formed via liquid-to-liquid phase transition in the vicinity of histone gene clusters (Duronio and Marzluff, 2017; Matera et al., 2009; Nizami et al., 2010). The U7 snRNP core is functionally incompetent, and its conversion into an active endonuclease capable of cleaving histone pre-mRNAs at the 3′ end is thought to occur only during the S-phase, concomitant with the activation of histone gene transcription by NPAT (Skrajna et al., 2016). This step depends on FLASH, a protein of 200 kDa (Yang et al., 2009), whose N-terminal coiled-coil domain interacts with the N-terminal region of Lsm11 (Aik et al., 2017; Yang et al., 2009). The resultant complex recruits the endonuclease module, a complex of three proteins: symplekin, CPSF100 and CPSF73 (Sun et al., 2020; Yang et al., 2013). In this complex, CPSF73 is the actual catalytic component directly responsible for hydrolyzing a phosphodiester bond in histone pre-mRNA (Dominski et al., 2005; Sun et al., 2020; Yang et al., 2020). The same complex is also used during cleavage coupled to polyadenylation, suggesting that the two eukaryotic 3′ end processing mechanisms share a common evolutionary origin (Dominski et al., 2005; Mandel et al., 2006).

In primary human cells, HLBs are formed in the vicinity of replication-dependent histone gene clusters on chromosomes 1 and 6 (Ghule et al., 2008; Ma et al., 2000; Zhao et al., 2000). The cluster on chromosome 6 is significantly larger, and HLBs assembled on this chromosome are detected by immunofluorescence (IF) microscopy throughout the interphase. HLBs that are associated with the smaller histone gene cluster on chromosome 1 are readily detectable only during the S-phase. As a result, primary human cells, depending on the phase of the cell cycle, contain two to four HLBs (Ma et al., 2000; Zhao et al., 2000). Transformed human cell lines, including HeLa cells, due to their aneuploidy and multiple chromosomal rearrangements typically contain more than four HLBs (Barcaroli et al., 2006; Bongiorno-Borbone et al., 2008).

We asked whether NPAT, FLASH and Lsm11 maintain their localization in HLBs in postmitotic muscle and neural cells that have ceased to divide and permanently exited the cell cycle, apparently eliminating the need to express replication-dependent histone genes. Our results demonstrate that in these cells, the three components of the histone gene expression machinery are still expressed but change their localization from nuclear to cytoplasmic, raising the possibility that they become repurposed for new functions specific for differentiated cells. We identified a specific region in Lsm11 that engages in various protein–protein interactions, likely controlling both the localization and function of Lsm11 and its complexes depending on the differentiation status of the cell. Finally, we discuss how our findings may help us understand the nature of the molecular defect in SMA, which selectively affects two functionally interdependent and terminally differentiated cell types: motor neurons and skeletal muscles.

## RESULTS

### Intracellular localization of exogenous Lsm11 in HeLa cells

**Fig. 1.**
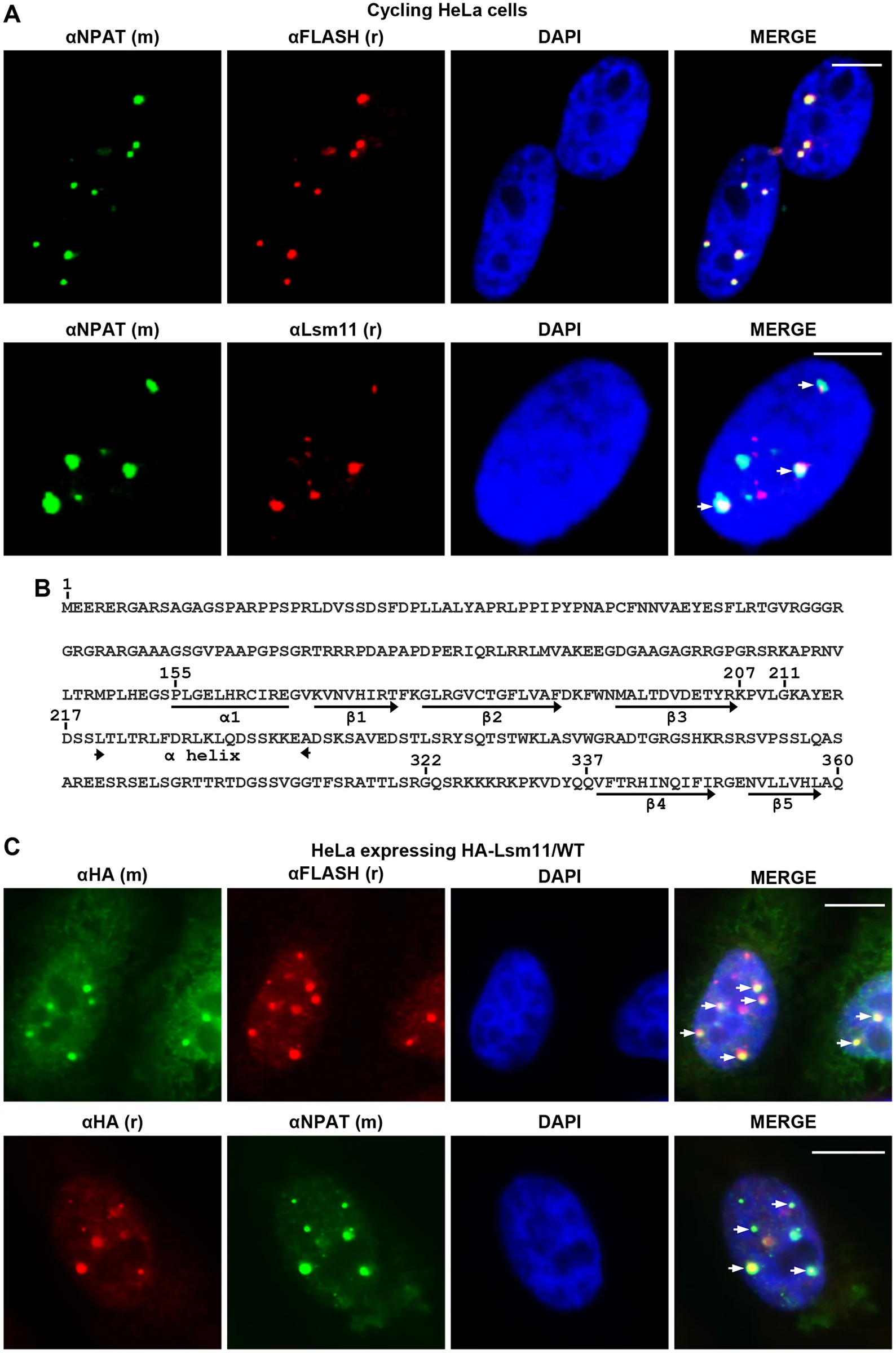
**Ectopically expressed HA-tagged WT Lsm11 localizes to HLBs.** (A) Indirect IF images of cycling HeLa cells co-stained with antibodies against NPAT and FLASH (top row) or NPAT and Lsm11 (bottom row). The NPAT antibody was of mouse origin (m) and was used together with a goat anti-mouse secondary antibody tagged with Alexa Fluor 488 (green signal). FLASH and Lsm11 were generated in rabbits (r) and were used together with a goat anti-rabbit secondary antibody tagged with Alexa Fluor 568 (red signal). The arrows indicate anti-Lsm11-stained foci that colocalize with HLBs detected by the anti-NPAT antibody. Nuclei were visualized by staining DNA with DAPI. Scale bars: 10 μM. (B) The sequence of the 360-residue human Lsm11. The N-terminal region encompasses amino acids 1-154. The Sm fold consists of a single α-helix (α1) and five β-strands (β1-β5) and encompasses amino acids 155-360. The internal loop (amino acids 207-337) that separates β3 and β4 contains a likely α-helix (indicated with arrows) and several conserved residues, including glycine 211 and aspartic acid 217. (C) Intracellular localization of WT Lsm11 stably expressed in HeLa cells, as analyzed by indirect IF. The protein contains an HA tag at the N-terminus and was detected by either a mouse (top row) or rabbit anti-HA antibody (bottom row). The HLBs were stained with anti-FLASH and anti-NPAT antibodies (top and bottom rows, respectively). The arrows indicate foci stained with anti-HA antibody that colocalize with HLBs. Scale bars: 10 μM.

We tested the suitability of several antibodies to analyze the intracellular localization of NPAT, FLASH and Lsm11 in HeLa cells by IF microscopy. In agreement with previous studies (Barcaroli et al., 2006; Bongiorno-Borbone et al., 2008; Ghule et al., 2008), a mouse monoclonal antibody against NPAT and a rabbit polyclonal antibody against FLASH demonstrated a perfect colocalization of these two proteins in nuclear HLBs (Fig. 1A, top panels). A rabbit antibody raised against Lsm11, a marker for the U7 snRNP core, also detected a variable number of nuclear foci, as previously reported (He et al., 2025). However, only some of them overlapped with HLBs detected by the anti-NPAT antibody (Fig. 1A, bottom panels, white arrows). The remaining foci likely represent CBs through which the U7 snRNP core transits prior to entering the HLBs (Matera et al., 2007).

Previous work showed that FLASH and the U7 snRNP core are targeted to HLBs independently, as two separate entities (Burch et al., 2011; Tatomer et al., 2016). FLASH and NPAT interact through their C-terminal domains and are predicted to act together to identify histone gene loci and to initiate the assembly of HLBs (Bucholc et al., 2020b; Skrajna et al., 2016; Yang et al., 2014). How the U7 snRNP core finds its way to these sites remains elusive. The most likely candidate to mediate this function is a large internal loop of an unknown role that spans amino acids 207-337 in Lsm11 and separates β-strands 3 (β3) and 4 (β4) of its Sm domain (Fig. 1B). A recombinant Lsm10/11 heterodimer lacking this loop is readily incorporated *in vitro* into the U7-specific ring, yielding a catalytically active U7 snRNP (Bucholc et al., 2020a; Sun et al., 2020; Yang et al., 2020). Thus, the deleted region of Lsm11 is not directly required for the U7 snRNP to function as a 3′ endonuclease, consistent with previous studies that assigned that role to the N-terminal extension of Lsm11 (Sun et al., 2020; Yang et al., 2009) (Fig. 1B; Fig. S11).

The internal loop is largely unstructured and evolutionarily diverse except for amino acids 219-235 that are highly conserved among vertebrate Lsm11 orthologs and likely fold into a helical structure, as suggested by AlphaFold predictions (Jumper et al., 2021). Highly conserved are also amino acids 207-213 that immediately follow β3, the acidic residue (D/E) at position 217, and a short segment at the end of the loop that directly precedes β4 (Fig. 1B; Fig. S1A). Interestingly, a single-point mutation substituting the highly conserved glycine 211 with serine was shown to contribute to the phenotype of the Aicardi-Goutières syndrome (AGS), a human disorder associated with the defective expression of replication-dependent histone genes (Uggenti et al., 2020). This observation indicates that at least some regions of the loop play an important role in the function of Lsm11 *in vivo*.

To determine if the internal loop is required for the proper subcellular localization of Lsm11 and its complexes, we used HeLa cells to stably express hemagglutinin (HA)-tagged wild-type (WT) Lsm11 and its four mutants: ΔL (lacking amino acids 211-332 of the loop), ΔH (lacking amino acids 219-235 of the α-helical structure), G211S (containing a substitution of glycine 211 with serine) and D217K (containing a substitution of aspartic acid 217 with lysine) (Fig. S1B). Western blotting with an anti-HA antibody demonstrated that HA-tagged Lsm11 variants were expressed at comparable levels in HeLa cells selected for G418 resistance (Fig. S2A; see also Fig. 4B, lanes 1-4). Intracellular localization of the ectopically expressed Lsm11 was analyzed by IF microscopy using both a mouse antibody and a rabbit anti-HA antibody, with the HLBs being detected by a rabbit anti-FLASH antibody and a mouse anti-NPAT antibody, respectively. Regardless of which antibody pair was used, HA-tagged WT Lsm11 was invariably detected in nuclear foci, with many of them overlapping with HLBs (Fig. 1C, top and bottom; see Fig. S2 for more images), hence resembling the staining pattern of endogenous Lsm11 visualized by an anti-Lsm11 antibody (Fig. 1A).

**Fig. 2.**
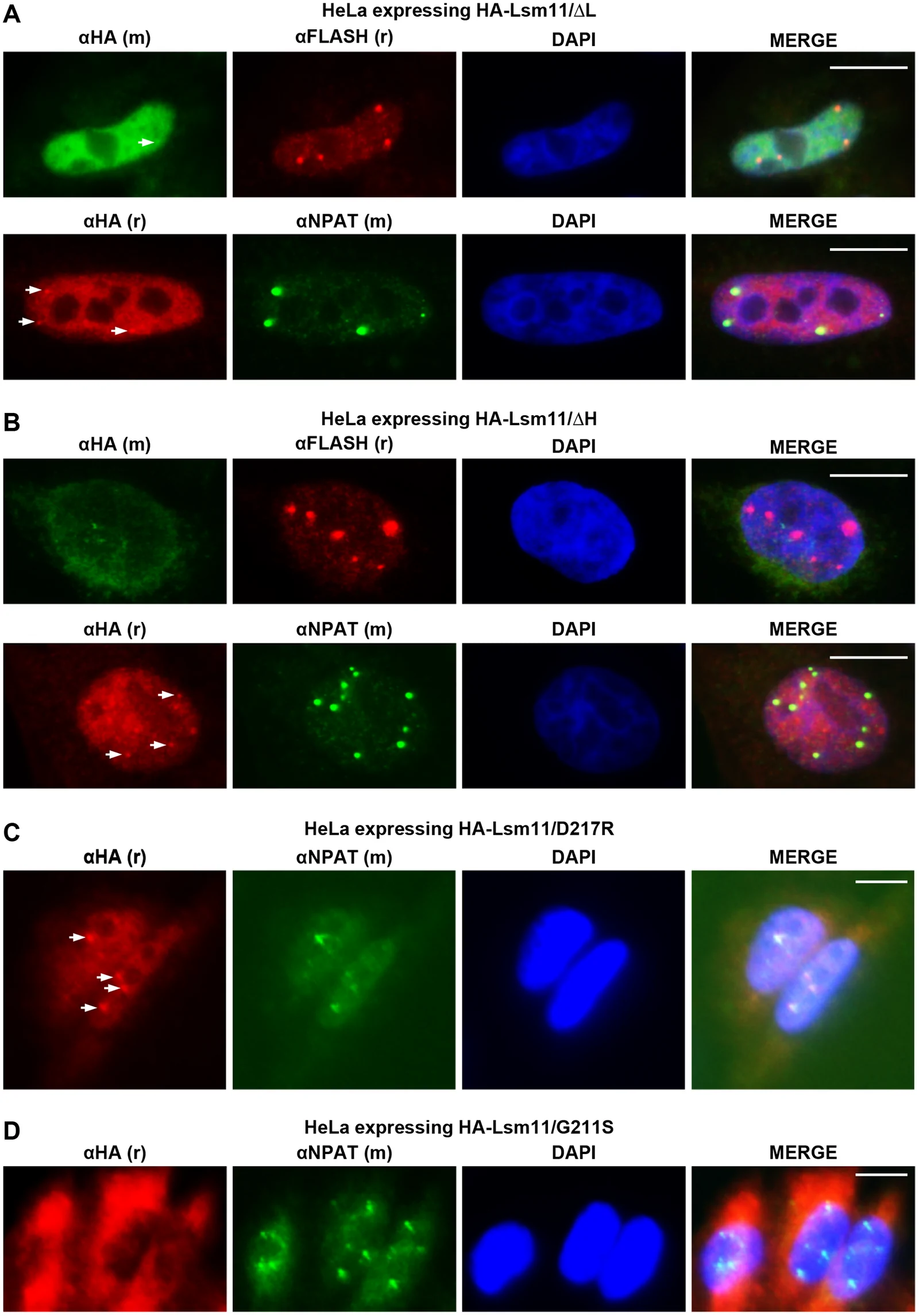
**Mutations within the internal loop of exogenous Lsm11 affect its localization to HLBs.** (A-D) Intracellular localization of the indicated HA-tagged Lsm11 loop mutants stably expressed in HeLa cells was analyzed by indirect IF. In panels A and B, Lsm11 mutants were stained using both the mouse (top row) and rabbit anti-HA antibodies (bottom row), with the HLBs being detected by anti-FLASH and anti-NPAT antibodies, respectively. In panels C and D, only the rabbit anti-HA antibody was used. Arrows indicate residual HLBs detected by the two anti-HA antibodies. Scale bars: 10 μM.

In contrast to the exogenous WT Lsm11, HA-tagged ΔL (Fig. 2A; Fig. S3) and ΔH mutant proteins (Fig. 2B; Fig. S4) failed to concentrate in HLBs and instead were detected in the nucleoplasm and occasionally in the cytoplasm. In some cells, both the mouse and rabbit anti-HA antibodies detected residual HLBs (Fig. 2A,B, arrows). A virtually WT staining pattern was observed for the D217R Lsm11, which was clearly targeted to HLBs (Fig. 2C, arrows; Fig. S5A). However, the presence of a relatively strong nucleoplasmic HA signal in some cells suggested a minor localization defect. On the other hand, a very severe localization defect was observed for HA-Lsm11 containing a different amino acid substitution, G211S, previously linked to the AGS (Uggenti et al., 2020). In most cells, this mutant Lsm11 was almost exclusively detected in the cytoplasm (Fig. 2D; Fig. S5B).

**Fig. 3.**
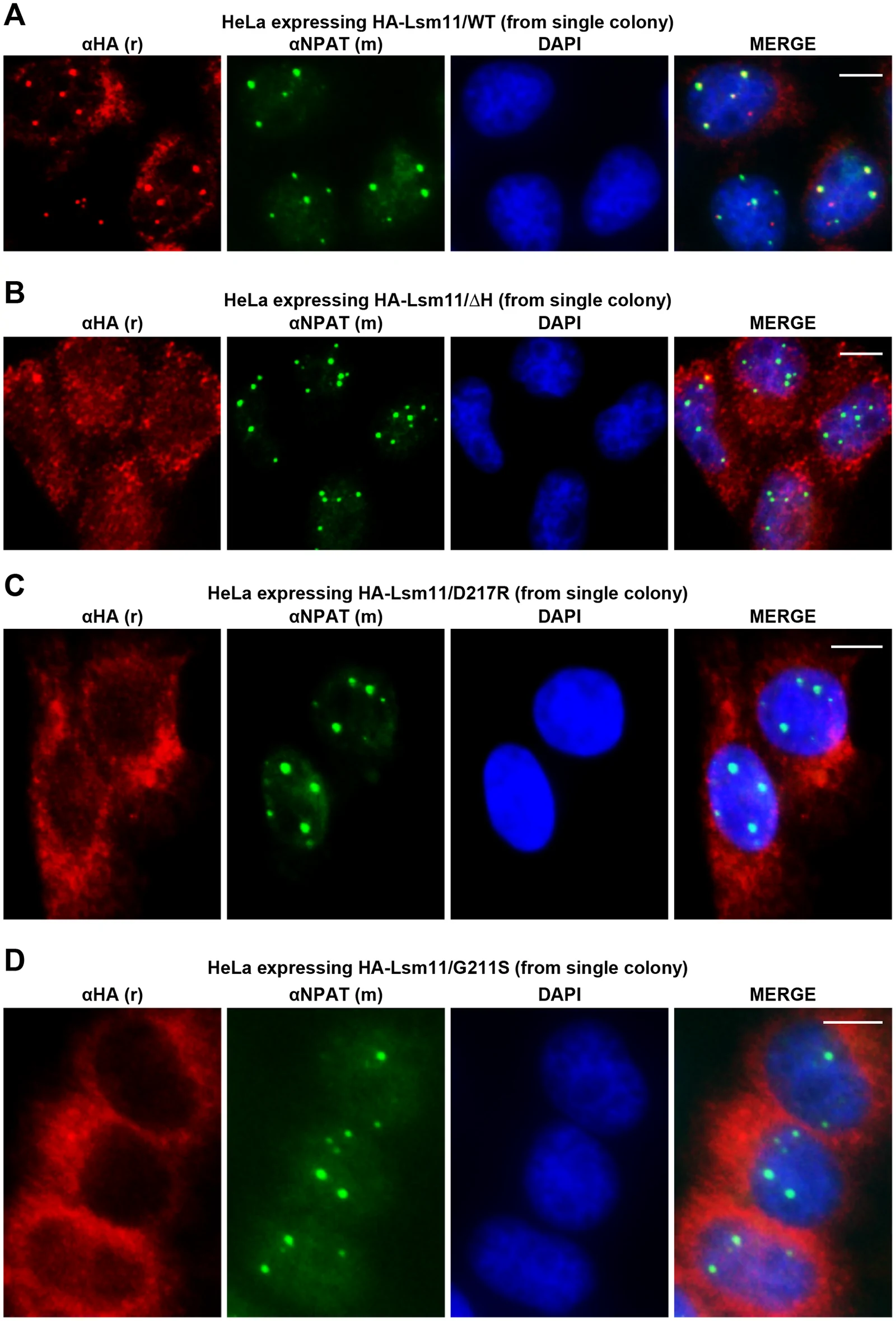
**Localization of exogenous Lsm11 in clonally selected cells**. (A-D) HeLa cells expressing the indicated HA-tagged variants of Lsm11 were cultured from individual G418-resistant colonies and used in IF experiments with a rabbit anti-HA antibody to detect HA-tagged Lsm11 and a mouse anti-NPAT antibody to detect HLBs. Scale bars: 10 μM.

In cell cultures maintained for a longer time in the presence of G418, the HA-tagged WT Lsm11 was invariably concentrated in nuclear foci, including HLBs, whereas all the HA-tagged Lsm11 mutants displayed a predominantly or exclusively cytoplasmic localization. The same cytoplasmic localization of Lsm11 mutants, including the D217R mutant, was observed in HeLa cultures initiated from individual G418-resistant colonies (Fig. 3B-D). Presumably, the nucleoplasmic localization of Lsm11 mutants negatively affects cell growth, providing a selective advantage to cells that developed a mechanism to retain mutant proteins in the cytoplasm. Importantly, prolonged clonal selection did not affect the localization of WT Lsm11, which was detected in HLBs (Fig. 3A). Altogether, our results clearly demonstrate that both larger deletions and point mutations within the conserved region of Lsm11 loop prevent Lsm11 from localizing to the nucleus and HLBs, the site of their activity in dividing cells.

### Incorporation of exogenous Lsm11 into the U7 snRNP core and binding partners of the Lsm11 loop

The incorrect localization of exogenous Lsm11 carrying loop mutations could result from their inability to support the *in vivo* assembly of the Sm ring around U7 snRNA via the pathway controlled by the SMN complex (Battle et al., 2006; Gruss et al., 2017). This step is required for the hypermethylation of the 5′ cap of U7 snRNA and subsequent import of the fully assembled U7 snRNP core to the nucleus (Fischer et al., 1993; Matera et al., 2007).

**Fig. 4.**
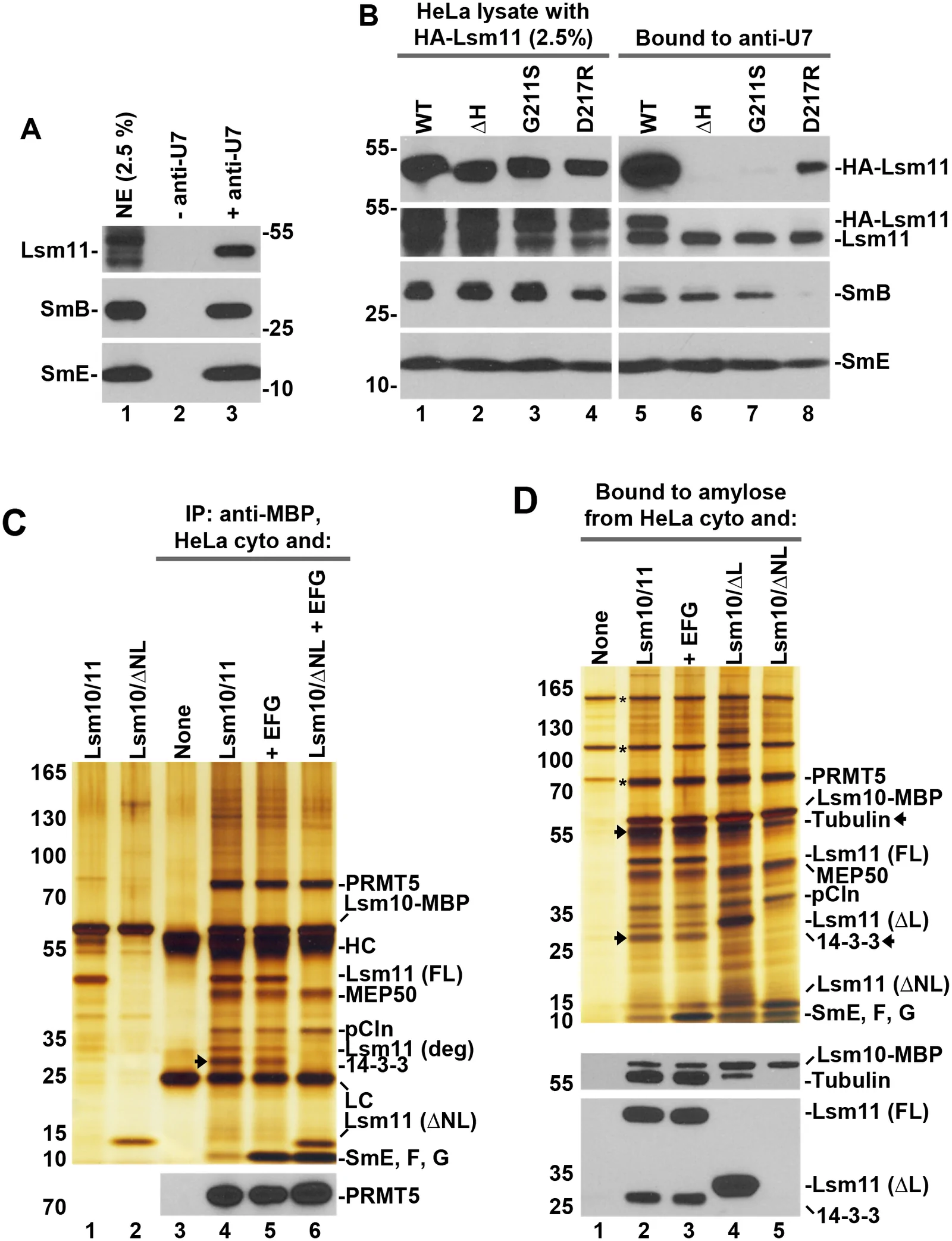
**Incorporation of exogenous Lsm11 into the U7 snRNP core and binding partners of the Lsm11 loop.** (A) An anti-U7 oligonucleotide complementary to nucleotides 2-17 of mammalian U7 snRNA specifically purifies U7 snRNP from a HeLa nuclear extract (NE), as judged by the presence of Lsm11, SmB and SmE in the precipitate (lane 3). No detectable amounts of these U7 snRNP subunits are purified in the absence of the oligonucleotide (lane 2). Lane 1 represents ∼2.5% of the NE used in each purification. (B) Lysates from HeLa cells stably expressing the indicated variants of HA-tagged Lsm11 were incubated with the anti-U7 oligonucleotide. The purified U7 snRNP was analyzed for the presence of both endogenous and exogenous Lsm11, using anti-HA and anti-Lsm11 antibodies, respectively, and the two other subunits, SmB and SmE (lanes 5-8). Lanes 1-4 represent ∼2.5% of the lysate used in each purification. (C,D) HeLa cytoplasmic proteins interacting with the Lsm10/11 heterodimer were purified by immunoprecipitation with anti-MBP antibody followed by their immobilization on protein A agarose beads (C) or by direct immobilization on amylose beads via MBP attached to Lsm10 (D). The purified proteins were detected by silver staining or western blotting (top and bottom panels, respectively). Lanes 1 and 2 in panel C contain aliquots of Lsm10/11 and Lsm10/ΔNL heterodimers used in the experiment. Lane 3 of panel C shows anti-MBP antibody and proteins of the HeLa cytoplasm bound to protein A beads in the absence of recombinant Lsm10/11. Lane 1 of panel D represents background proteins of the HeLa cytoplasm bound to amylose beads in the absence of recombinant Lsm10/11. Silver-stained bands containing potential Lsm10/11 interactors are indicated with arrows.

To test whether the HA-tagged WT Lsm11 and its various mutants become incorporated *in vivo* into an Sm ring around U7 snRNA, we used an anti-U7 oligonucleotide that is complementary to the 5′ region of mammalian U7 snRNA and contains biotin at the 3′ end (Skrajna et al., 2018; Smith et al., 1991). This oligonucleotide allows for efficient and specific purification of U7 snRNP from various sources, including human and mouse nuclear extracts (Fig. 4A), as previously demonstrated (Yang et al., 2013). HeLa cells stably expressing WT, ΔH, G211S and D217R Lsm11 were lysed and shown by western blotting with an anti-HA antibody to contain comparable levels of HA-tagged Lsm11 variants (Fig. 4B, lanes 1-4, top panel; Fig. S2A). The lysates were incubated with the anti-U7 oligonucleotide, and the bound U7 snRNP was immobilized on streptavidin beads and analyzed using an anti-HA antibody to detect HA-tagged exogenous Lsm11 and anti-Lsm11 antibody to detect both the tagged Lsm11 and endogenous Lsm11 lacking the tag. In addition, we used antibodies against two common U7 snRNP subunits, SmB and SmE, that are required to complete the assembly of the Sm ring around U7 snRNA.

As determined by using the anti-HA and anti-Lsm11 antibodies, U7 snRNP purified from WT Lsm11-expressing HeLa cells contained a readily detectable HA-tagged WT Lsm11, and its amount matched the amount of the untagged endogenous Lsm11 (Fig. 4B, lane 5, two top panels). The purified material also contained SmB and SmE. Thus, the exogenous WT Lsm11 is successfully incorporated into the U7 snRNP core, consistent with the detection of this protein by IF in HLBs. Vastly different results were obtained with cell lysates from HeLa cells expressing HA-tagged ΔH and G211S. These exogenous Lsm11 mutants were not present in the purified U7 snRNP, although endogenous untagged Lsm11 was readily detectable, indicating a successful purification (Fig. 4B, lanes 6 and 7). As detected by the anti-HA antibody, a small amount of Lsm11 containing a D217R substitution was incorporated into the U7 snRNP (Fig. 4B, compare lanes 5 and 8), supporting the notion that this substitution is not as disruptive as the ΔH and G211S mutations, consistent with the IF experiments. Note that the D217R lysate was more diluted than the other lysates tested (Fig. 4B, lane 4), as illustrated by a relatively weak signal for SmB protein (Fig. 4B, lane 4). Altogether, our results suggest that mutations of conserved loop residues prevent the incorporation of Lsm11 into the Sm ring.

The inability of the loop mutants to support the assembly of the U7 snRNP core prompted us to look for proteins that may recognize this part of Lsm11. We incubated a WT Lsm10/11 heterodimer with a cytoplasmic extract prepared from HeLa cells and precipitated the bound proteins using anti-MBP (maltose-binding protein) antibody that detects the MBP tag attached to Lsm10. Silver staining of the material immobilized on protein A beads, besides Lsm10 and Lsm11, revealed the presence of three methylosome components, PRMT5, Mep50 and pICln (Fig. 4C, top panel, lane 4), consistent with previous results (Yang et al., 2023). The identity of PRMT5 was confirmed by western blotting (Fig. 4C, bottom panel, lane 4). In the same sample, silver staining visualized a 30 kDa band (Fig. 4C, lane 4, arrow) that was not contributed by the heterodimer itself (Fig. 4C, lane 1) or the cytoplasm and the antibody (Fig. 4C, lane 3). The 30 kDa band was also detected in a sample additionally containing an SmE/F/G heterotrimer (Fig. 4C, lane 5), which is known to form transient complexes with an Lsm10/11 heterodimer (Chari et al., 2008; Zhang et al., 2011). Importantly, this band was absent when a WT heterodimer was replaced with Lsm10/ΔNL lacking both the internal loop and the N-terminal region in Lsm11 (Fig. 4C, lane 6).

In a similar set of experiments, we omitted the anti-MBP antibody and directly immobilized the heterodimer and bound cytoplasmic proteins on amylose beads via the MBP attached to Lsm10. The same band of ∼30 kDa was again visualized by silver staining, and the presence of the SmE/F/G trimer had no effect on its intensity (Fig. 4D, top panel, lanes 2 and 3, arrow). One additional band was also visible at about 55 kDa (Fig. 4D, top panel, lane 2, arrow). This band comigrates with the heavy chain of immunoglobulin and a degradation product of Lsm10-MBP, precluding its identification in the immunoprecipitation experiment shown in Fig. 4C. The 30 kDa band was not detected when Lsm10/ΔL and Lsm10/ΔNL heterodimers were used (Fig. 3D, lanes 4 and 5), indicating that the interaction requires the loop sequence. Under the same conditions, the 55 kDa band appeared significantly weaker, although the presence of an Lsm10-MBP degradation product in the same region and excess of Lsm10/ΔL used in the sample partially obscure this result (see below). As expected, binding of the three subunits of the PRMT5 methylosome was not affected by the two deletions in Lsm11.

Mass spectrometry identified the 30 and 55 kDa bands as the 14-3-3 and tubulin family proteins, respectively. Western blotting confirmed these identities and clearly demonstrated that the binding of 14-3-3 and tubulin proteins to the heterodimer critically depends on the internal loop of Lsm11, as illustrated by having no interaction (14-3-3) or only a very weak interaction (tubulin) when Lsm10/ΔL was used instead of WT Lsm10/11 (Fig. 4D, bottom panels, lane 4). In the case of tubulin, a smaller role may be additionally played by the N-terminal region of Lsm11, with the complete disruption of the interaction requiring deleting both regions (Fig. 4D, bottom panels, lane 5).

### Localization of NPAT, FLASH and Lsm11 in muscle cells

We reasoned that the internal loop of Lsm11 may function as a unique module to control the localization and function of Lsm11 and its complexes depending on whether cells are continuously dividing, thus replicating chromatin during each consecutive S-phase, or terminally differentiated, thus being independent of the cyclical supply of histone proteins. To test this possibility, we asked whether the key components of the histone gene expression machinery, NPAT, FLASH and Lsm11, are enriched in HLBs of postmitotic muscle and neural cells that have permanently exited the cell cycle.

**Fig. 5.**
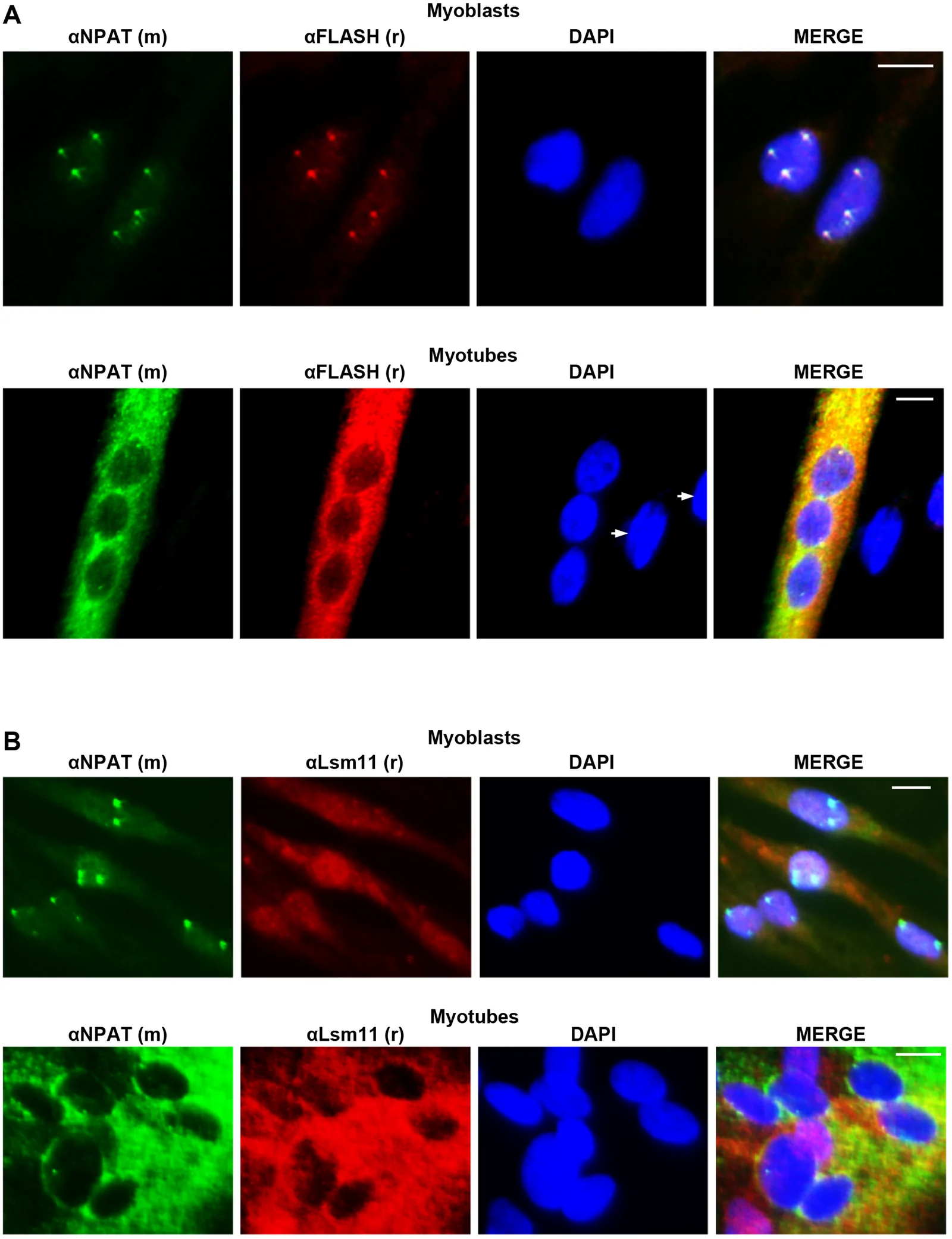
**Localization of NPAT, FLASH and Lsm11 during differentiation of muscle cells.** (A) Intracellular localization of NPAT and FLASH in constantly cycling LHCN-M2 myoblasts (top row) and postmitotic multinuclear LHCN-M2 myotubes (bottom row). (B) Intracellular localization of NPAT and Lsm11 in constantly cycling LHCN-M2 myoblasts (top row) and postmitotic multinuclear LHCN-M2 myotubes (bottom row). In both panels, nuclei were stained with DAPI. Scale bars: 10 μM.

We first used the LHCN-M2 line of immortalized human skeletal myoblasts that were maintained as an exponentially dividing culture or induced for 7 days to differentiate into nondividing and multinucleated myotubes, as previously described (Zhu et al., 2007). In constantly cycling LHCN-M2 myoblasts, NPAT and FLASH colocalized in two larger and two smaller HLBs (Fig. 5A, top row; Fig. S6A), corresponding to histone gene loci on chromosomes 6 and 1, respectively, thus resembling other primary diploid cell lines (Ma et al., 2000). Strikingly, in all postmitotic multinucleated myotubes, both NPAT and FLASH were excluded from the nuclei, producing instead a readily detectable signal in the cytoplasm (Fig. 5A, bottom row; Fig. S6B). In the same culture, we also observed mononuclear cells, where FLASH and NPAT were undetectable (Fig. 5A, bottom row, arrows), or rare binuclear cells, where NPAT was detected exclusively in the cytoplasm, with FLASH being detected in both the nucleoplasm and cytoplasm (Fig. S7A). These cells may represent early stages of differentiation prior to or immediately after myoblast fusion. Finally, in some other LHCN-M2 cultures that were unsuccessfully induced for differentiation and failed to yield multinuclear myotubes, cells often displayed the nucleoplasmic localization of NPAT and FLASH, with residual HLBs still being detected in some of them (Fig. S7B). These various staining patterns seem to suggest that in myoblasts triggered for differentiation, HLB disassembly is followed by the leakage of NPAT and FLASH to the nucleoplasm, their degradation and *de novo* synthesis and retention in the cytoplasm of postmitotic myotubes.

The intracellular localization of Lsm11, a subunit of the U7 snRNP core, was monitored using the same rabbit anti-Lsm11 antibody that detects nuclear foci in HeLa cells (Fig. 1A, bottom row). Surprisingly, in constantly dividing LHCN-M2 myoblasts, in a sharp contrast to constantly dividing HeLa cells, this antibody detected a diffuse nucleoplasmic localization of Lsm11 (Fig. 5B, top row), indicating that in these cells, the U7 snRNP core does not concentrate in HLBs. Even more notably, in some cells, Lsm11 was present in both the nucleoplasm and the cytoplasm or almost exclusively in the cytoplasm (Fig. S8A). This unusual staining pattern of Lsm11 may reflect the propensity of cycling LHCN-M2 cells to readily exit the cell cycle and to differentiate into myotubes and suggests that the leakage of the U7 snRNP core from HLBs to the nucleoplasm precedes HLB disassembly. Importantly, the signal for Lsm11 in postmitotic multi-nuclear LHCN-M2 myotubes was exclusively detected in the cytoplasm (Fig. 5B, bottom row; Fig. S8B), with the nuclei having the appearance of dark holes, resembling the staining pattern of NPAT and FLASH in the same cell culture.

We conclude that at least three components of the histone gene expression machinery, NPAT, FLASH and Lsm11, change their localization from the nuclear HLBs to the cytoplasm during the differentiation of skeletal muscle cells.

### Localization of NPAT, FLASH and Lsm11 in neural cells

**Fig. 6.**
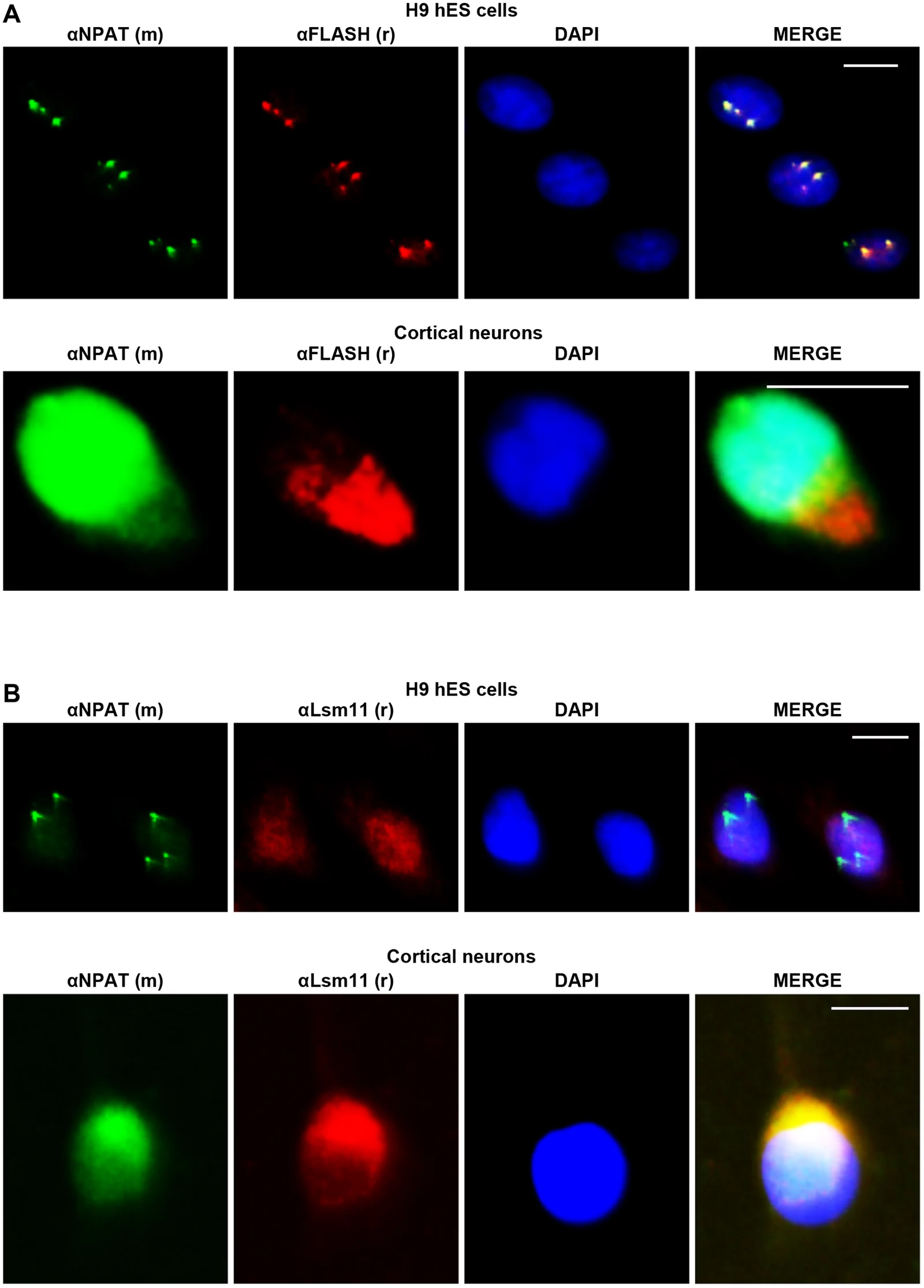
**Localization of NPAT, FLASH and Lsm11 during differentiation of neural cells.** (A) Intracellular localization of NPAT and FLASH in constantly cycling H9 hES cells (top row) and postmitotic cortical neurons (bottom row). (B) Intracellular localization of NPAT and Lsm11 in constantly cycling H9 hES cells (top row) and postmitotic cortical neurons (bottom row). In both panels, nuclei were stained with DAPI. Scale bars: 10 μM.

The same antibodies were used to analyze the localization of NPAT, FLASH and Lsm11 in mitotically active H9 human embryonic stem (hES) cells stably expressing the human Neurogenin 2 (NGN2) transcription factor and postmitotic cortical neurons. Again, in exponentially dividing cells, NPAT and FLASH perfectly colocalized in two brighter HLBs (histone gene locus on chromosome 6 pair) and two smaller HLBs (histone gene locus on chromosome 1 pair) (Fig. 6A, top row). In a cell culture treated for 5 days to differentiate H9 hES cells into cortical neurons and to eliminate mitotically active cells, the anti-NPAT and anti-FLASH antibodies failed to detect HLBs. In virtually all postmitotic cells of the treated culture, the antibodies revealed instead a diffuse localization of NPAT and FLASH, with each protein being captured in the process of leaving the nucleus and localizing toward a single, less frequently double, cytoplasmic protrusion (Fig. 6A, bottom row; Fig. S9A). The advancement of the nuclear exit differed among individual cells and between the two proteins. In many cells, FLASH was almost entirely redistributed to this protrusion, with NPAT clearly lagging and remaining in a significant portion of the nucleus. These observations suggest that FLASH and NPAT move to the cytoplasm as separate entities, contrasting with their invariably perfect colocalization in HLBs of dividing cells, and that FLASH may leave the nucleus prior to NPAT.

In all constantly dividing H9 hES cells, the anti-Lsm11 antibody revealed a dispersed localization of Lsm11 in the nucleus (Fig. 6B, top row), resembling the localization of this protein in dividing LHCN-M2 cells and contrasting with its localization to HLBs in dividing HeLa cells (Fig. 1A, bottom row). In postmitotic cortical neurons, Lsm11 gradually exited the nucleus and moved toward one (Fig. 6B, bottom row), or less frequently two cytoplasmic protrusions, mimicking the behavior of NPAT and FLASH (Fig. S9B). At least in some cells, Lsm11 appeared to exit the nucleus ahead of NPAT. It is therefore possible that Lsm11 as part of the U7 snRNP core and FLASH are exported to the cytoplasm together as a single complex, independently of NPAT.

### Yeast two-hybrid screens to identify new binding partners of Lsm11 and FLASH

**Fig. 7.**
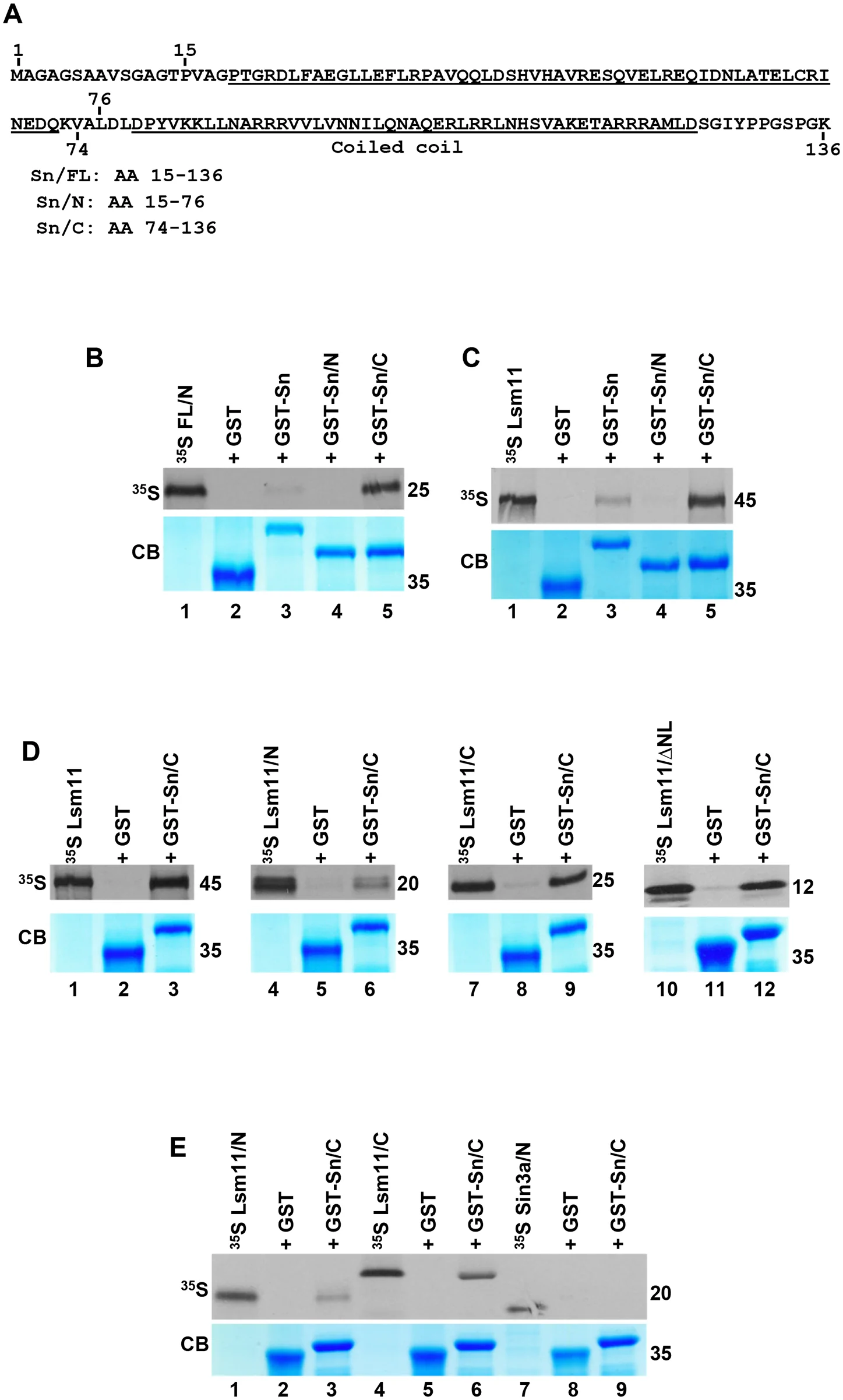
**Biochemical interaction of FLASH and Lsm11 with Snapin.** (A) The sequence of human Snapin. The two helical regions are underlined, and the terminal amino acids of the three Snapin fragments, Sn, Sn/N and Sn/C, are indicated at the top of the sequence. (B-E) GST alone or GST fused to each of the three Snapin fragments was incubated with the indicated ^35^S-labeled proteins, purified on glutathione beads and separated by SDS-PAGE. ^35^S-labeled and GST-tagged proteins were detected by radiography (top panels) or Coomassie Blue (CB) staining (bottom panels), respectively. Aliquots of ^35^S-labeled proteins (∼20% of the amount used in each binding assay) are shown in lane 1 of panels B and C, lanes 1, 4, 7 and 10 of panel D and lanes 1, 4 and 7 of panel E.

We previously used the yeast two-hybrid system and a HeLa cDNA library to look for proteins that interact with Lsm11 and identified the N-terminal region of FLASH as the most frequently isolated protein prey (Yang et al., 2009). We searched for additional binding partners of Lsm11 and the N-terminal region of FLASH that could provide initial clues on how these two proteins function in postmitotic cells that do not express replication-dependent histone genes. For this purpose, we screened these two baits against several human two-hybrid cDNA libraries, including a universal library that encodes proteins expressed in various tissues, organs and developmental stages. Strikingly, SNARE-associated protein (Snapin) was identified as the most frequently isolated binding partner for both Lsm11 and the N-terminal region of FLASH. Human Snapin is a 136-amino-acid protein previously implicated in various aspects of autophagy, lysosomal functions and synaptic vesicle recycling in neurons (Cai et al., 2010; Ilardi et al., 1999; Tian et al., 2005). Residues 19-72 and 79-125 are predicted to fold into helical structures, with the downstream helix potentially forming a coiled-coil structure (Ilardi et al., 1999) (Fig. 7A). Interestingly, of the multiple Snapin cDNA clones isolated with either bait, the vast majority encoded the entire C-terminal helix and only small portions of the N-terminal helix, with the full-length Snapin being isolated rarely.

We tested the interaction between Snapin and either Lsm11 or the N-terminal FLASH biochemically using a GST binding assay. Human Snapin encompassing amino acids 15-136, thus lacking the unstructured N-terminal hydrophobic region, was cloned with a GST tag at the N-terminus and expressed in bacteria (GST-Sn). In addition, each of the two Snapin helices was expressed separately, yielding GST-Sn/N (amino acids 15-76) and GST-Sn/C (amino acids 74-136). The recombinant proteins were incubated with the following ^35^S-labeled proteins expressed in a rabbit reticulocyte lysate (Figs S1A and S10A): FL/N (amino acids 1-139 of human FLASH), Lsm11 (full-length human Lsm11, amino acids 1-360), Lsm11/N (amino acids 1-167 of human Lsm11), Lsm11/C (amino acids 153-360 of human Lsm11), Lsm11/ΔNL (amino acids 153-210 and 323-360 of human Lsm11) and Sin3a/N (amino acids 1-139 of human Sin3a). GST-tagged recombinant proteins were incubated with ^35^S-labeled proteins, immobilized on glutathione beads, separated by SDS-PAGE and visualized by Coomassie Blue staining to detect GST-tagged proteins and by autoradiography to evaluate the amount of interacting ^35^S-labeled proteins.

The N-terminal region of FLASH, previously shown to interact via its amino acids 100-130 with Lsm11 (Yang et al., 2009, 2011), also binds the C-terminal half of Snapin containing the second helix (Fig. 7B, lane 5), but not the N-terminal half containing the first helix (Fig. 7B, lane 4) or GST alone (Fig. 7B, lane 2). Only a residual binding was detected with Snapin encompassing amino acids 15-136 (GST-Sn), which contains both helices (Fig. 7B, lane 3), consistent with the two-hybrid screens. The same binding pattern was observed for Lsm11 (Fig. 7C, lanes 3-5).

To identify which part of Lsm11 interacts with GST-Sn/C, we tested the three shorter Lsm11 variants. The N-terminal half of Lsm11 (Lsm11/N) showed only a relatively weak interaction (Fig. 7D, lanes 4-6, Fig. 7E, lanes 1-3). This result was surprising since amino acids 25-40 of Lsm11 interact with the coiled-coil homodimer of FLASH, and we expected that the same region mediates the interaction with the structurally similar region of Snapin. Instead, the interaction largely involves the C-terminal half of Lsm11, which adopts the Sm fold with a single α-helix and five antiparallel β-strands (Fig. 7D, lanes 7-9, Fig. 7E, lanes 4-6). As determined using Lsm11/ΔNL, no major role in this interaction is played by the internal loop of Lsm11 that separates β-strands 3 and 4 (Fig. 7D, lanes 10-12). These results suggest that the C-terminal helix of Snapin may collectively recognize both the N-terminal region of Lsm11 and its Sm fold, with the internal loop making no significant contribution.

The fact that the C-terminal helix of Snapin interacts with both FLASH and Lsm11 could potentially indicate that it is prone to nonspecific contacts. This possibility prompted us to use an unrelated sequence derived from the N-terminal region of human Sin3a (Sin3a/N) instead of FLASH and Lsm11. Importantly, the GST pulldown assay showed that GST-Sn/C failed to interact with Sin3a/N (Fig. 7E, lane 9), although in the same assay, it consistently bound the C-terminal half of Lsm11 (Fig. 7E, lanes 4-6). We also note that multiple screens with the same libraries and baits other than Lsm11 and FLASH never yielded Snapin as a positive clone, arguing against a general propensity of this protein to engage in nonspecific interactions.

While the interaction of Lsm11 and the N-terminal region of FLASH with Snapin requires further validation, it potentially implicates these two components of the histone gene expression machinery in cytoplasmic events linked to membrane fusion, including the release of neurotransmitter-containing synaptic vesicles at neuronal synapses (Cai et al., 2010; Ilardi et al., 1999; Tian et al., 2005). Intriguingly, the N-terminal region of FLASH displays a strong ability to form disulfide bonds (Fig. S10) (Aik et al., 2017), a feature characteristic of extracellular or membrane-associated proteins, further supporting the notion that this typically nuclear protein may also play a role beyond the nucleus.

## DISCUSSION

The expression of replication-dependent histone genes is controlled by two dedicated factors: NPAT that activates transcription of histone genes and U7 snRNP that co-transcriptionally cleaves the resulting pre-mRNAs at the 3′ end, yielding translationally active mRNAs. Both events are cell cycle regulated and occur only during the S-phase concomitantly with DNA replication, providing large quantities of histone proteins for *de novo* chromatin assembly. The core of U7 snRNP consists of U7 snRNA and a surrounding ring of five Sm subunits shared with the spliceosomal snRNPs and two unique subunits, Lsm10 and Lsm11 (Khusial et al., 2005; Schumperli and Pillai, 2004). FLASH is a regulatory subunit of U7 snRNP and interacts with the N-terminal region of Lsm11 to recruit the endonuclease module consisting of symplekin, CPSF100 and CPSF73, forming a functionally competent U7 snRNP (Dominski et al., 2005; Sun et al., 2020; Yang et al., 2020). In cycling cells, NPAT and components of the U7 snRNP concentrate in HLBs, nuclear condensates formed in the vicinity of histone gene clusters via liquid-to-liquid phase transition, increasing both the efficiency and coordination of transcription and 3′ end processing in histone gene expression (Duronio and Marzluff, 2017; Nizami et al., 2010).

The mechanism that restricts the formation of HLBs to the vicinity of histone gene clusters remains unknown, although an important clue may come from the fact that NPAT and FLASH tightly interact with each other through their C-terminal domains (Bucholc et al., 2020b; Yang et al., 2014). According to one model, this interaction renders each protein functionally inert, hence blocking the expression of histone genes in the G1-phase but at the same time facilitating the recognition of histone gene loci and the assembly of HLBs (Skrajna et al., 2016). Hyperphosphorylation of NPAT by CDK2/cyclin E at the G1/S-phase transition (Ma et al., 2000; Zhao et al., 1998, 2000) could disrupt the inert complex, simultaneously activating the transcription of replication-dependent histone genes and U7 snRNP-mediated 3′ end processing by the liberated NPAT and FLASH, respectively (Skrajna et al., 2016).

### The role of the internal loop in Lsm11

How the U7 snRNP core is targeted to the HLBs is unknown (Burch et al., 2011). Lsm11 is the most characteristic subunit of the U7-specific Sm ring and the largest protein of the Sm/Lsm family, significantly exceeding in size the remaining members of the family due to the presence of a long N-terminal region of 154 amino acids and a large internal loop of 131 amino acids located within its Sm fold (Khusial et al., 2005; Schumperli and Pillai, 2004). While the main function of the N-terminal region is to form a platform with FLASH for recruiting the endonuclease module to the U7 snRNP core, the role of the long internal loop in Lsm11, including residues highly conserved in all vertebrates, is unknown. Interestingly, as determined by structural studies (Sun et al., 2020) and Alfa-Fold predictions, the N-terminal extension and the internal loop are located at the opposing sides of the Sm ring, consistent with these two regions of Lsm11 playing functionally distinct roles (Fig. S11).

In this study, we demonstrated that deleting the internal loop from Lsm11 ectopically expressed in HeLa cells results in the inability of the mutant protein to support the assembly of the U7 snRNP core *in vivo* and hence to localize to the nucleus and HLBs. The same effect was caused by a smaller deletion that removed a highly conserved helical structure of the loop or by point mutations within the adjacent highly conserved region, including G211S mutation. This single-amino-acid substitution has been linked to the AGS, a rare genetic disorder that results from a defective expression of replication-dependent histone genes (Uggenti et al., 2020), supporting our conclusion about the importance of the internal loop of Lsm11.

To understand how the loop may contribute to the function of Lsm11, we looked for its binding partners in extracts from HeLa cells. We showed that the Lsm10/11 heterodimer, but not its mutant lacking the loop within Lsm11, associates with proteins of the 14-3-3 family. The human genome encodes seven highly similar 14-3-3 proteins, which are best characterized for their ability to recognize phosphorylated protein motifs and hence to regulate a plethora of cellular processes, including signal transduction, transcription, cell cycle progression, apoptosis, and protein trafficking (Obsilova and Obsil, 2022). The 14-3-3 proteins also engage in phosphorylation-independent interactions, acting in a chaperone-like manner (Sluchanko and Gusev, 2017). Interestingly, one of the key activities of 14-3-3 proteins is to regulate the coalescence of various proteins into phase-separated condensates, a function of potential relevance for Lsm11 and its ability to target the U7 snRNP core to HLBs (Segal et al., 2023; Stoecklin et al., 2004).

In addition to the 14-3-3 family, we identified proteins of the tubulin family as other binding partners of Lsm11. Again, their association vastly depended on the internal loop. Tubulin proteins by forming microtubules are essential components of the cytoskeleton and regulate cell shape, intracellular trafficking, axon development and synaptic function in neurons, among other functions (McKenna et al., 2023). It is unclear whether 14-3-3 and tubulin proteins interact with the same or different regions of the internal loop. Their interaction with Lsm11 may also be mediated by only one of the two proteins, with the other protein being recruited indirectly. Finally, it is possible that the interacting interface includes regions of Lsm10 that are adjacent to the internal loop of Lsm11. Additional studies are required to determine whether Lsm11 interacts with 14-3-3 and tubulin proteins *in vivo* and to identify other binding partners that recognize its internal loop. Nevertheless, an attractive possibility is that the 14-3-3 and tubulin proteins function to deliver the Lsm10/11 heterodimer to cytoplasmic sites specialized in the assembly of the U7 snRNP core and to ultimately promote its localization to the nuclear HLBs for further assembly steps and 3′ end processing of histone pre-mRNAs.

### HLB components change localization in postmitotic cells

We reasoned that 14-3-3 and tubulin proteins may additionally function by rerouting Lsm11 and its complexes to cell compartments other than HLBs in postmitotic cells. These cells have permanently exited the cell cycle and do not rely on the massive supply of replication-dependent histone proteins during each consecutive S-phase and hence on the assembly of HLBs. To address this possibility, we analyzed the fate of HLBs in two different cell types, depending on their differentiation status. As expected, in continuously dividing human skeletal LHCN-M2 myoblasts (progenitors of postmitotic muscle cells) and H9 hES cells with inducible expression of the NGN2 transcription factor (progenitors of cortical neurons), both NPAT and FLASH were detected in four HLBs per cell, consistent with the presence of four histone gene loci in human primary cell lines. Surprisingly, Lsm11 localized to the nucleoplasm of these cells, contrasting with its HLB localization in continuously dividing HeLa cells. Thus, Lsm11 as part of the U7 snRNP core is not actively recruited to HLBs in LHCN-M2 myoblasts and in the engineered H9 embryonic stem cells, potentially reflecting their strong tendency to differentiate. This distinct localization of Lsm11 further confirms that the U7 snRNP core and FLASH, despite being capable of forming a complex together, are targeted to HLBs as two independent entities, as proposed (Burch et al., 2011; Skrajna et al., 2016).

In postmitotic LHCN-M2 myotubes and cortical neurons generated after several days of differentiation, HLBs were dismantled and NPAT, FLASH and Lsm11 were detected in the cytoplasm. Interestingly, the manner of how these proteins exit the nucleus seems to differ between the two cell types. Our results suggest that early during the differentiation of dividing myoblasts into postmitotic and multinuclear myotubes, HLBs are undetectable, and both NPAT and FLASH become dispersed throughout the nucleoplasm. This step is likely followed by the degradation of NPAT and FLASH and their *de novo* synthesis and retention in the cytoplasm. We hypothesize that the U7 snRNP core is also degraded in the nucleus, with the Lsm10/11 heterodimer being *de novo* synthesized and retained in the cytoplasm as one of the intermediate complexes that precede the assembly of the U7 snRNP core and its licensing for nuclear import. These intermediates include the ring-shaped 6S-like complex of Lsm10/11, the SmE/F/G heterotrimer and pICln (Chari et al., 2008; Paknia et al., 2016; Yang et al., 2023; Zhang et al., 2011), where both Lsm11 and SmE are symmetrically dimethylated within their N-terminal regions (Yang et al., 2023). The purpose of this unique modification remains unknown, and one possibility is that it may serve specific functions in the cytoplasm of postmitotic cells.

A different relocalization mechanism for NPAT, FLASH and the U7 snRNP core containing Lsm11 likely operates in postmitotic cortical neurons. The HLBs already lacking the U7 snRNP core are disassembled, mimicking their fate in LHCN-M2 myotubes, and the three proteins dispersed in nucleoplasm are gradually redistributed toward a typically single cytoplasmic protrusion. The most visible effect of this unidirectional movement is the formation of a protein-free nuclear pole located opposite the cytoplasmic protrusion. In most postmitotic cortical neurons, FLASH and Lsm11 seem to relocalize ahead of NPAT, although it is unknown whether this is the obligatory order of events. This observation suggests that the interaction of NPAT and FLASH via their C-terminal regions is disrupted, triggering the disassembly of the HLBs and the association of liberated FLASH with Lsm11 of the U7 snRNP core.

### Potential functions of components of the histone gene expression machinery in postmitotic cells and links to SMA

The fact that NPAT, FLASH and Lsm11 reside in the cytoplasm of postmitotic cells raises the possibility that they become repurposed in these cells for functions other than histone gene expression. Previous studies documented that several other cell cycle regulators, including cyclins and cyclin-dependent kinases, perform various noncanonical functions in postmitotic cells, indicating that functional repurposing of ‘unemployed’ proteins is a common theme in cell differentiation (Frank and Tsai, 2009; Hydbring et al., 2016).

What might be the role of components of the histone gene expression machinery in the cytoplasm of postmitotic muscle and neural cells? One possibility comes from our two-hybrid screens, which identified the C-terminal half of Snapin containing an extended helix capable of forming coiled-coil structures as a binding partner of both FLASH and Lsm11. Surprisingly, full-length Snapin containing an additional helix within the N-terminal half interacts only weakly with FLASH and Lsm11. This observation suggests that the N-terminal helix functions as a regulatory domain blocking the ability of the C-terminal helix to interact with each of the two proteins. Snapin was previously shown to interact with the SNARE complex (Ilardi et al., 1999), the key component of the membrane fusion pathway (Yoon and Munson, 2018), and was implicated in regulating synaptic vesicle release and recycling (Cai et al., 2010; Ilardi et al., 1999; Sudhof and Rothman, 2009; Tian et al., 2005). This function of Snapin potentially links relocalized components of the histone gene expression machinery to events that occur near the plasma membrane, including neuronal synapses. In support of this possibility, FLASH has a strong tendency to form intermolecular disulfide bonds within its N-terminal coiled-coil dimer, indicative of extracellular localization.

The assembly of the U7 snRNP core requires the SMN protein (Pillai et al., 2003; Tisdale et al., 2013, 2022), including its Tudor domain that recognizes symmetrically dimethylated arginines (Cote and Richard, 2005; Courchaine et al., 2021; Tripsianes et al., 2011). Deficiency of SMN, which is also required for the assembly of spliceosomal snRNPs (Battle et al., 2006; Gruss et al., 2017; Neuenkirchen et al., 2008), results in a common genetic syndrome, SMA. At the clinical level, SMA is characterized by selective death of motor neurons and progressive muscle wasting, although it remains unclear how these tissue-specific effects can be reconciled with the general housekeeping function of SMN in snRNP biogenesis (Beattie and Kolb, 2018; Monani, 2005). Intriguingly, the SMN protein was recently shown to also regulate the assembly of the SNARE complex at neuromuscular junctions (NMJs) (Kim et al., 2023; Matera, 2025). A tempting hypothesis is that the ‘unemployed’ subunits of U7 snRNP and other components of the histone gene expression machinery may act together with Snapin and the remodeling activity of SMN to facilitate feedback communication between motor neurons and muscle fibers, two main types of terminally differentiated cells in animals, hence maintaining the integrity and functionality of NMJs. While hypothetical, this reasoning suggests future research avenues in studying SMA, potentially providing new insights into the pathophysiology of this neuromuscular syndrome.

## MATERIALS AND METHODS

### Antibodies

The rabbit anti-Lsm11 antibody used in western blotting was generated by Pocono Rabbit Farm (Canadensis, PA, USA) using the N-terminal region of *Danio rerio* Lsm11 as the immunogen. This antibody cross-reacted with both the mouse and human Lsm11 orthologs. The following commercial antibodies were used in this study: mouse anti-NPAT (Santa Cruz Biotechnology), rabbit anti-FLASH (A301-662A-1, Bethyl Laboratories), rabbit anti-HA (clone C29F4, Cell Signaling Technology), mouse anti-HA (66006-2-Ig, Proteintech), rabbit anti-Lsm11 (PA5-31324, Invitrogen), and mouse anti-MBP (66003-1-Ig, Proteintech). For western blotting, primary antibodies were typically diluted 2500-fold. Exposed films were processed using Adobe Photoshop 2025 software.

### Expression of recombinant proteins

WT and mutant Lsm10/11 heterodimers were expressed in insect cells using the baculovirus system (Sun et al., 2021). cDNA encoding full-length or appropriate fragments of Snapin was cloned into the pET42a vector (Novagen), and GST-tagged Snapin variants were expressed in *Escherichia coli* and purified on Ni-NTA agarose beads (Qiagen) using standard protocols.

### ^35^S protein labeling and GST pulldown assay

FL/N and various fragments of Lsm11 were synthesized *in vitro* by the coupled transcription-and-translation (TnT) system (Promega) using rabbit reticulocyte lysates and ^35^S methionine, as suggested by the manufacturer. The GST binding assay was carried out, as previously described (Skrajna et al., 2016; Yang et al., 2011). The ^35^S-exposed autoradiograms were processed using Adobe Photoshop 2025 software.

### Yeast two-hybrid screens

Various Mate and Plate cDNA libraries (Takara Bio Inc.) were screened according to the manufacturer’s protocol and as previously described (Yang et al., 2009).

### Stable expression of exogenous HA-tagged Lsm11 in HeLa cells

HeLa cells were purchased from the American Type Culture Collection and cultured in DMEM/10% FBS medium as monolayers, following the manufacturer’s recommendations. Cell cultures were expanded by allowing only a limited number of doublings to avoid contamination and frozen in multiple aliquots for subsequent use. cDNA fragments encoding WT Lsm11 and its loop mutants were cloned into HA-pcDNA3 immediately downstream of the HA tag-encoding region, and 2.5 μg of the resulting DNA constructs was transfected into HeLa cells using Lipofectamine 3000 (Invitrogen), as recommended by the manufacturer. 2 days following transfection, the medium was supplemented with G418 at 0.5 mg/ml and changed every 2 days. The G418-resistant cells were pooled or clonally selected from individual colonies, and the resultant cultures were used to analyze the expression and localization of the HA-tagged Lsm11 variants by western blotting and IF, respectively.

### IF experiments

Control or stably transfected HeLa cells were transferred onto a glass coverslip and incubated overnight in DMEM/10% FBS medium at 37°C. The following day, the cells were rinsed with PBS, fixed with PBS/4% paraformaldehyde for 10 min at room temperature, rinsed again with PBS, and permeabilized with PBS/0.1% Triton X-100 for 10 min. Following three brief washes with the same buffer, the glass coverslip was soaked in PBS/5% normal goat serum for 30 min. Primary antibodies were diluted 500-fold in the same buffer, applied to the coverslip and incubated at room temperature for 60 min. After the incubation, the coverslip was washed three times (10 min per wash) with PBS/0.1% Triton X-100. Next, the coverslip was incubated 60 min at room temperature with a solution of 5% goat serum in PBS containing an appropriate secondary antibody diluted 3000-fold (a goat anti-mouse secondary antibody tagged with Alexa Fluor 488 or a goat anti-rabbit secondary antibody tagged with Alexa Fluor 568). The coverslip was washed three times (10 min per wash) with PBS/0.1% Triton X-100, followed by one brief wash in PBS. The cells were treated for 5 min at room temperature with PBS containing DAPI at 0.5 μg/ml, followed by three brief washes with PBS. The coverslip was mounted onto microscope slides using Crystal/Mount mounting medium (Biomeda Corp.), air-dried and viewed using Keyence BZ-X800 Analyzer.

### Cell differentiation

LHCN-M2 cells (Zhu et al., 2007) were obtained from Evercyte and cultured by allowing only a limited number of doublings to avoid cross-contamination and kept frozen at −80°C in multiple aliquots for subsequent use. After thawing, the cells were plated on gelatinized glass coverslips in a six-well dish using 2 ml of a growth medium containing DMEM/M199 (Gibco) mixed in a 4:1 ratio, 15% FBS, 20 mM HEPES, 0.03 μg/ml zinc sulfate, 1.4 μg/ml vitamin B12, 55 ng/ml dexamethasone, 2.5 ng/ml hepatocyte growth factor and 10 ng/ml basic fibroblast growth factor. The cells were grown at 37°C as undifferentiated myoblasts in a humidified incubator in the presence of 5% CO_2_. When the culture reached 100% confluence, the growth medium was removed, the cells were rinsed twice with PBS, and myogenic differentiation was initiated by adding serum-free medium containing DMEM/M199 (Gibco) mixed in a 4:1 ratio, 20 mM HEPES, 0.03 μg/ml zinc sulfate, 1.4 μg/ml vitamin B12, 10 μg/ml insulin and 100 μg/ml apo-transferrin. The medium was changed every second day, and after 7 days of differentiation, the coverslips were used for IF experiments, as described above for HeLa cells. H9 hES cells expressing the human NGN2 transcription factor under a tetracycline-inducible promoter were maintained on hES cell-qualified Matrigel in Stem Flex medium and differentiated into cortical neurons, as described (https://www.protocols.io/view/indi-transcription-factor-ngn2-differentiation-of-n2bvj3owblk5/v1). At day 0, the cells (∼70% confluent) were split and treated with the induction medium containing doxycycline (1 μg/ml) and ROCK inhibitor (Y-27632, 10 μM). Fresh induction medium lacking the ROCK inhibitor was used on day 1. Between days 2 and 3, the cells were split again and maintained in the neuronal maturation medium lacking DMEM/F-12 but containing BrainPhys, doxycycline (1 μg/ml), 5-fluoro-2′-deoxyuridine (0.5 μM), and uridine (0.5 μM). In the following days, a second volume of fresh neuronal maturation medium was added to the culture every 3-4 days, with 5-fluoro-2′-deoxyuridine and uridine being omitted after day 7.

### HeLa cytoplasmic extracts

Cytoplasmic extracts were prepared from HeLa S3 cells grown as a suspension culture, as previously described (Sun et al., 2021; Yang and Dominski, 2025).

### NP40 lysates from HeLa cells

HeLa cells were collected by spinning, rinsed with PBS and lysed in NP40 lysis buffer (40 mM Tris pH 7.5, 150 mM NaCl, 0.5% NP40), as previously described (Yang and Dominski, 2025).

### Purification of U7 snRNP by an antisense oligonucleotide

U7 snRNP was purified from NP40 lysates by an antisense 2′-*O*-methyl oligonucleotide (anti-U7) complementary to nucleotides 2-17 of human U7 snRNA and containing biotin at the 3′ end, as described (Yang et al., 2013). Briefly, a whole-cell NP40 lysate from HeLa cells (500 μl) was mixed with 1.5 volume of a buffer containing 40 mM HEPES (pH 7.9), 150 mM NaCl, and 4.5 mM MgCl_2_ and rotated for 60 min at 4°C with 0.5 μg of the anti-U7 oligonucleotide. The sample was spun down for 10 min at 13,500×***g***, and then the supernatant was loaded onto 25 μl of streptavidin beads (Sigma) and rotated at 4°C for 60 min. The beads were gently spun, rinsed twice and rotated for an additional 60 min with a buffer containing 40 mM HEPES (pH 7.9), 150 mM NaCl, 2.5 mM MgCl_2_ and 0.2% NP40. A fraction of each sample was separated by electrophoresis in a 4%-12% SDS-PAGE, and the bound proteins were analyzed by western blotting with antibodies specific to individual components of the U7 snRNP.

## Supplementary Material

10.1242/biolopen.062755_sup1 Supplementary information
