# Supplementary material for: Are components of the histone gene expression machinery functionally repurposed in terminally differentiated cells?
Source: Biol Open. 2026 Aug 14;15(8):bio062755. doi: 10.1242/bio.062755 (PMC13552480; doi:10.1242/bio.062755)
Supplement: Supplementary file 1 [file biolopen-15-062755-s1.pdf]

A

```
 1
MEERERGARSAGAGSPARPPSPRLDVSSDSFDPLLALYAPRLPPIPYPNAPCFNNVAEYESFLRTGVRGGGR

GRGRARGAAAGSGVPAAPGPSGRTRRRPDAPAPDPERIQRLRRLMVAKEEGDGAAGAGRRGPGRSRKAPRNV
         153              167                                       211
LTRMPLHEGSPLGELHRCIREGVKVNVHIRTFKGLRGVCTGFLVAFDKFWNMALTDVDETYRKPVLGKAYER
217 220            234
DSSLTLTRLFDRLKLQDSSKKEADSKSAVEDSTLSRYSQTSTWKLASVWGRADTGRGSHKRSRSVPSSLQAS
    ➧      α helix      ➧          322                                 360
AREESRSELSGRTTRTDGSSVGGTFSRATTLSRGQSRKKKRKPKVDYQQVFTRHINQIFIRGENVLLVHLAQ
```

Lsm11: AA 1-360
Lsm11/N: AA 1-167
Lsm11/C: AA 153-360
Lsm11/ΔL: internal loop (AA 211-322) deleted
Lsm11/ΔH: internal helix (AA 220-234) deleted
Lsm11/ΔNL: AA 153-210 and AA 323-360
Lsm11/G211S: G211 mutated to S
Lsm11/D217R: D217 mutated to R

B

```
Hs   23   RLDVSSDSFDPLLALYAPRLPPIPYPNAPCFNNVAEYESFLRTGVRGGGRGRGRARGAAA   82
          +LD+ SD FDPLLALY+P++P +PYPN  CFNN+AEYESF++     GGRGR
Dr   50   KLDIRSDKFDPLLALYSPQVP-LPYPNIKCFNNIAEYESFMK-----GGRGR--------   95

Hs   83   GSGVPAAPGPSGRTRRRPDAPAPDPERIQRLRRLMVAKEEGDGAAGAGRRGPGRSRKAPR   142
               A P    +  R+       DPERI+RL+RLMV      +    G+G + P R RK  +
Dr   96   -----AKPENVEKRLRKAQKGKADPERIERLKRLMVNNPVPEEGEGSGVKRPPRQRKTAK   150

Hs   143  NVLTRMPLHEGSPLGELHRCIREGVKVNVHIRTFKGLRGVCTGFLVAFDKFWNMALTDVD   202
          NVLTRMPLH GSPLG+L+RC++E ++V VHIRTFKGLRGVC+GF+VAFDKFWN+A+ DVD
Dr   151  NVLTRMPLHTGSPLGQLNRCVQEKIRVKVHIRTFKGLRGVCSGFVVAFDKFWNLAMVDVD   210
              207
Hs   203  ETYRKPVLGKAYERDSSLTLTRLFDRLKLQDSSKKEADSKSAVE----DSTLSRYSQTST   258
          ETYR+P+LG+A    + +LT+TRLF++LK+Q+++      + SAVE     D  L     ++ +
Dr   211  ETYREPLLGQALYHEKALTITRLFEKLKVQETA-----ALSAVERKQGDKLLKSDPRSGS   265

Hs   259  WKLASVWGRADTGRGSHKRSRSVPSSLQASAREESRSELSGRTTRTDGSSVGGTFSRATT   318
           +L      R+D+     H + +    S +   S                        T SR
Dr   266  SQLKDQSRRSDS---RHNKEKPELSEIDKS-----------------------TSSR---   296
                        337
Hs   319  LSRGQSRKKKRKPKVDYQQVFTRHINQIFIRGENVLLVHLAQ   360
              + +K + +PKV+Y +V TRH+NQ+FIRGENVLLV++ Q
Dr   297  ----EMQKPESRPKVEYGRVHTRHVNQLFIRGENVLLVNIQQ   334
```

Fig. S1. A. The sequence of Lsm11 and the list of Lsm11 mutants used in this study. Amino acids located at the boundaries of the deleted regions and those substituted with other amino acids are numbered over the sequence. The internal loop is underlined, and the position of the presumptive α-helix is indicated with two arrows facing each other. **B.** BLAST alignment of amino acid sequences for human (top) and *Danio rerio* (bottom) Lsm11 orthologues to identify conserved residues within the loop (underlined).

Biology Open • Supplementary information

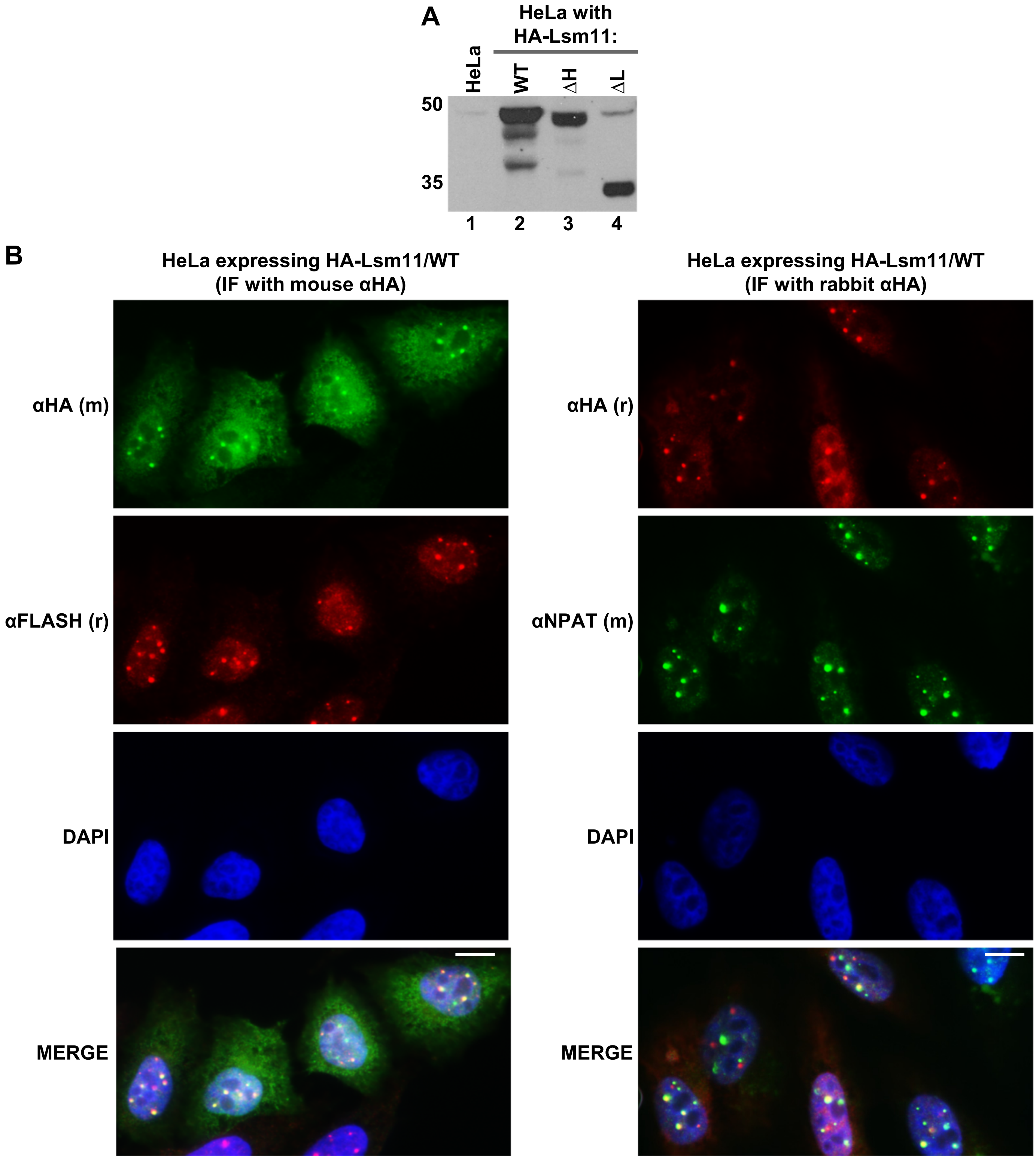

Fig. S2. A. HeLa cells transfected with plasmids encoding the indicated HA-tagged Lsm11 variants and conferring G418 resistance were grown in the presence of the antibiotic for several days. The resistant cells were pooled, lysed and analyzed for the expression of HA-tagged Lsm11 by Western blotting using an anti-HA antibody. A lysate from non-transfected HeLa cells is shown in lane 1. **B.** Intracellular localization of HA-tagged WT Lsm11 stably expressed in HeLa cells was analyzed by either a mouse (m, left column) or rabbit anti-HA antibody (r, right column). HLBs were detected using anti-FLASH or anti-NPAT antibodies, respectively. Scale bars represent 10 μM.

Biology Open • Supplementary information

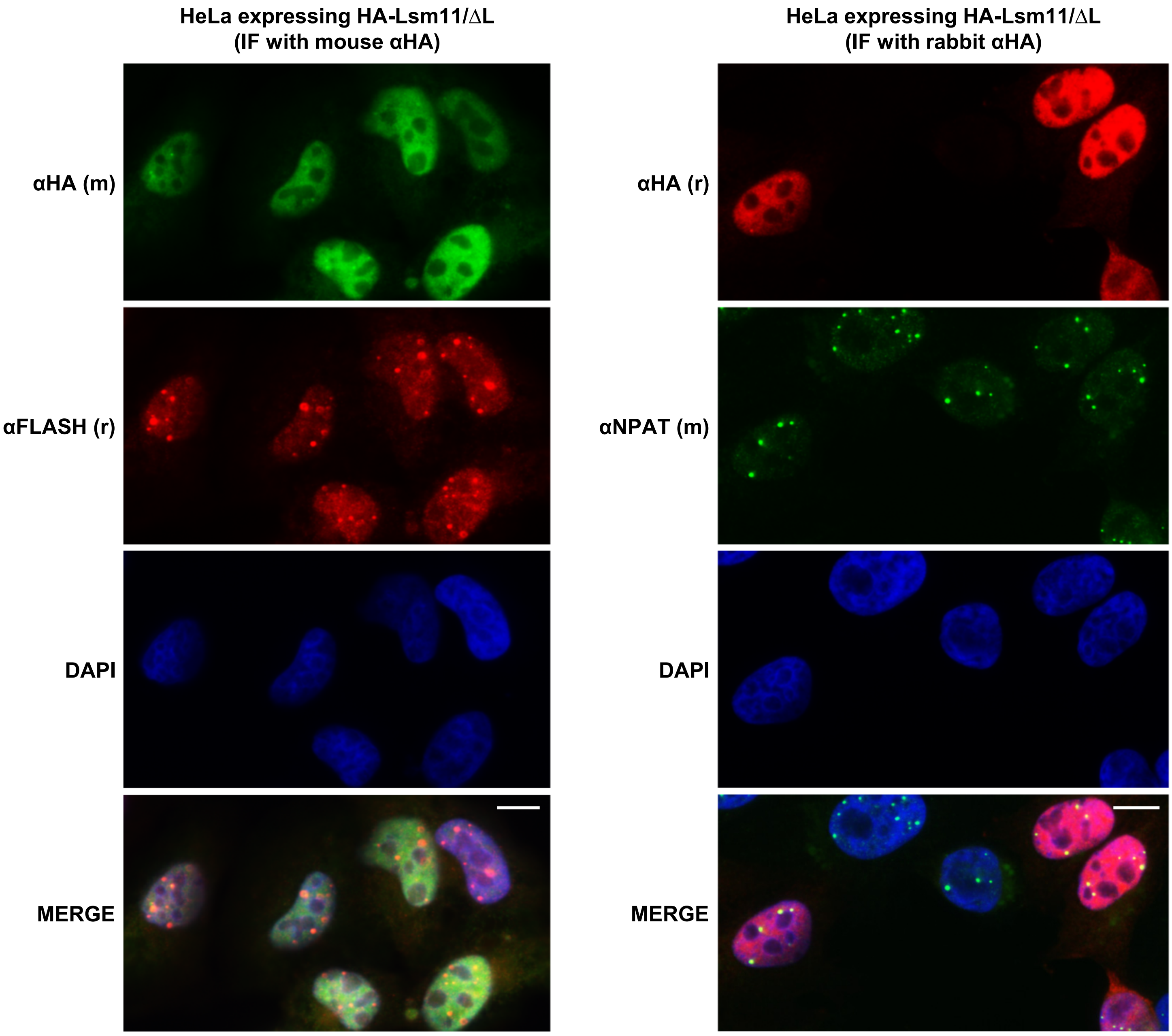

Fig. S3. Intracellular localization of HA-Lsm11/ΔL stably expressed in HeLa cells was analyzed using either a mouse (m, left column) or rabbit anti-HA antibody (r, right column). HLBs were detected using anti-FLASH or anti-NPAT antibodies, respectively. Scale bars represent 10 μM.

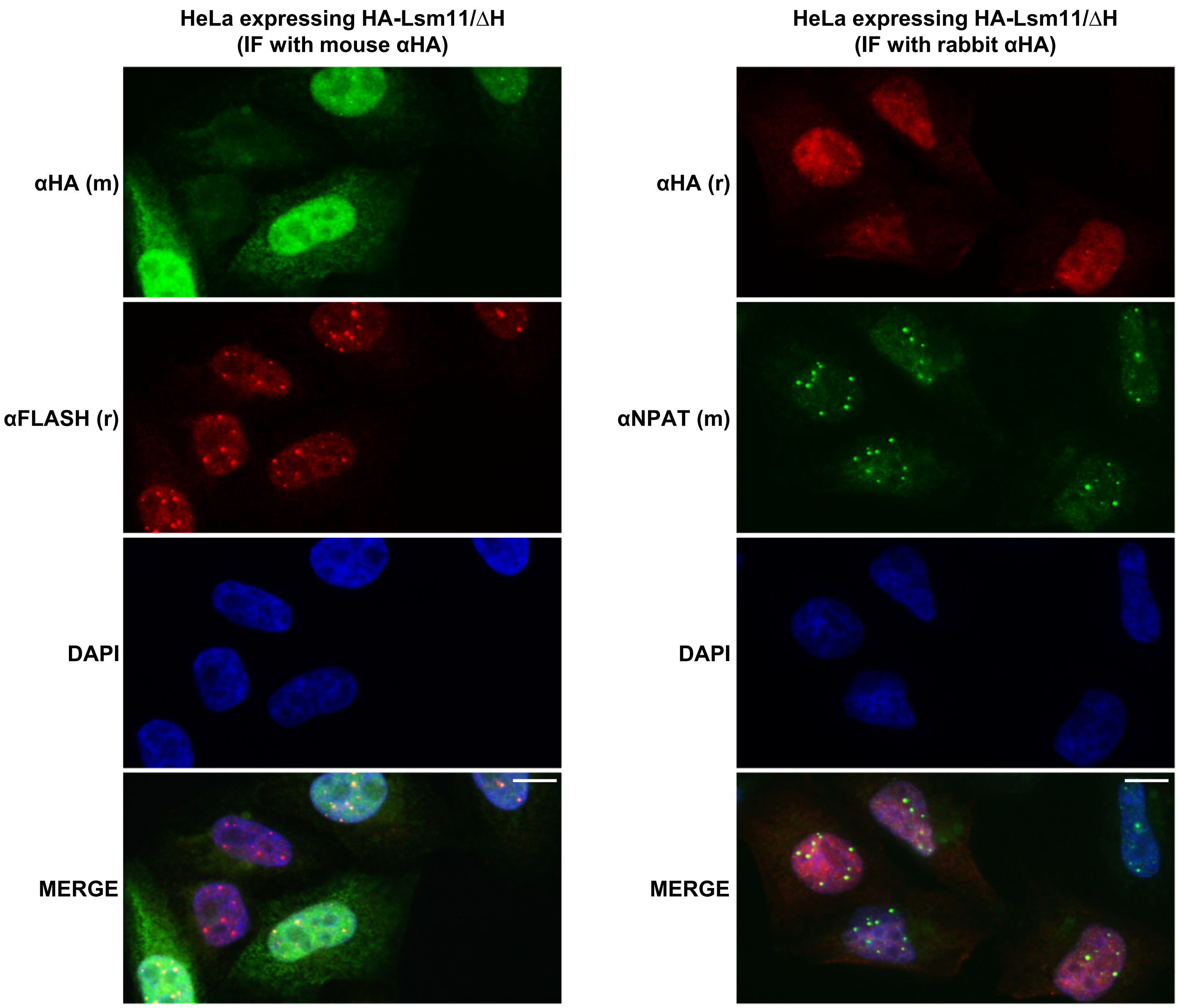

Fig. S4. Intracellular localization of HA-Lsm11/ΔH stably expressed in HeLa cells was analyzed using either a mouse (m, left column) or rabbit anti-HA antibody (r, right column). HLBs were detected using anti-FLASH or anti-NPAT antibodies, respectively. Scale bars represent 10 µM.

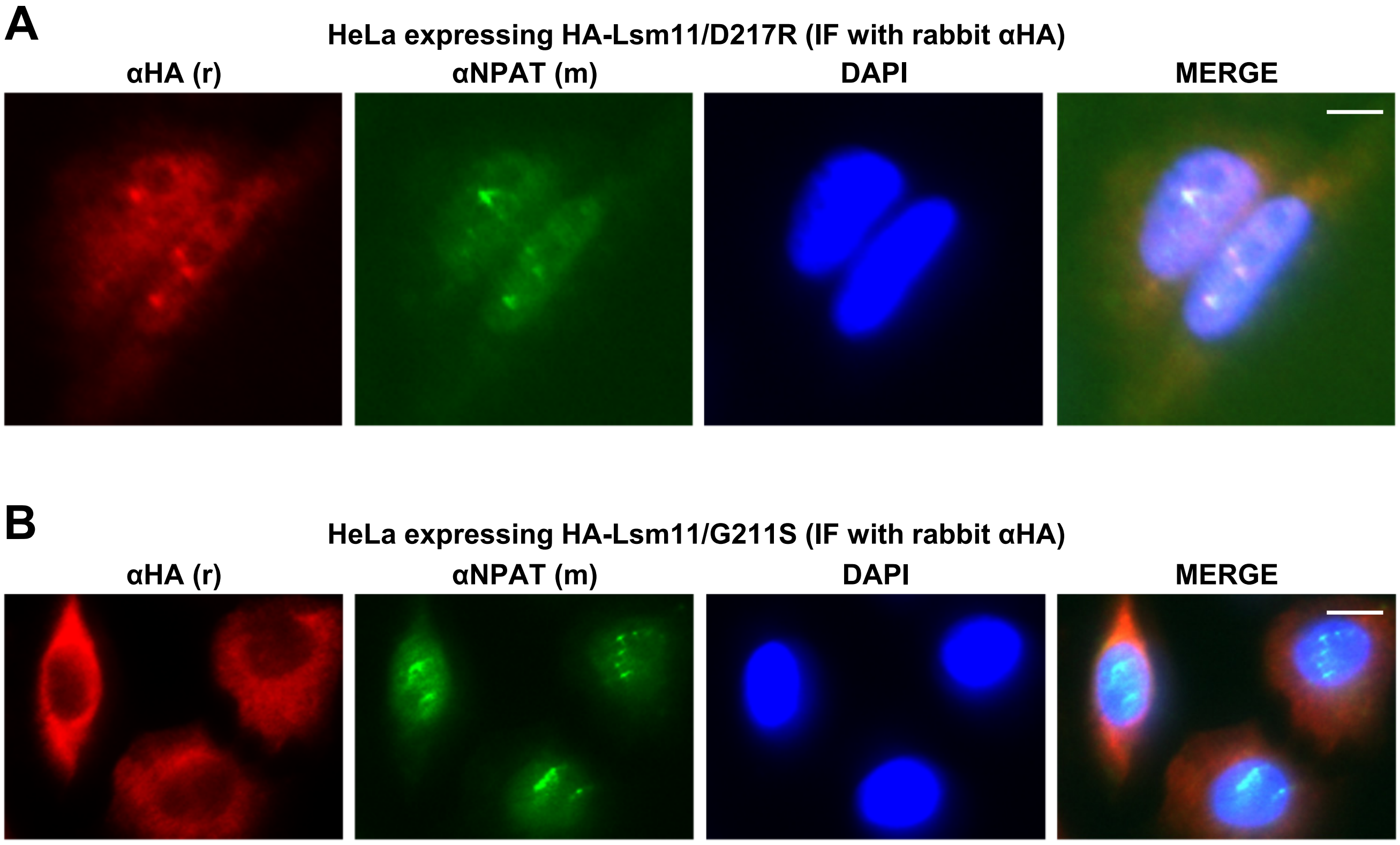

Fig. S5. A and B. Intracellular localization of HA-Lsm11/D217R (panel A) and HA-Lsm11/G211S (panel B) mutants stably expressed in HeLa cells was analyzed using a rabbit anti-HA antibody (r). HLBs were detected using an anti-NPAT antibody. Scale bars represent 10 μM.

Biology Open • Supplementary information

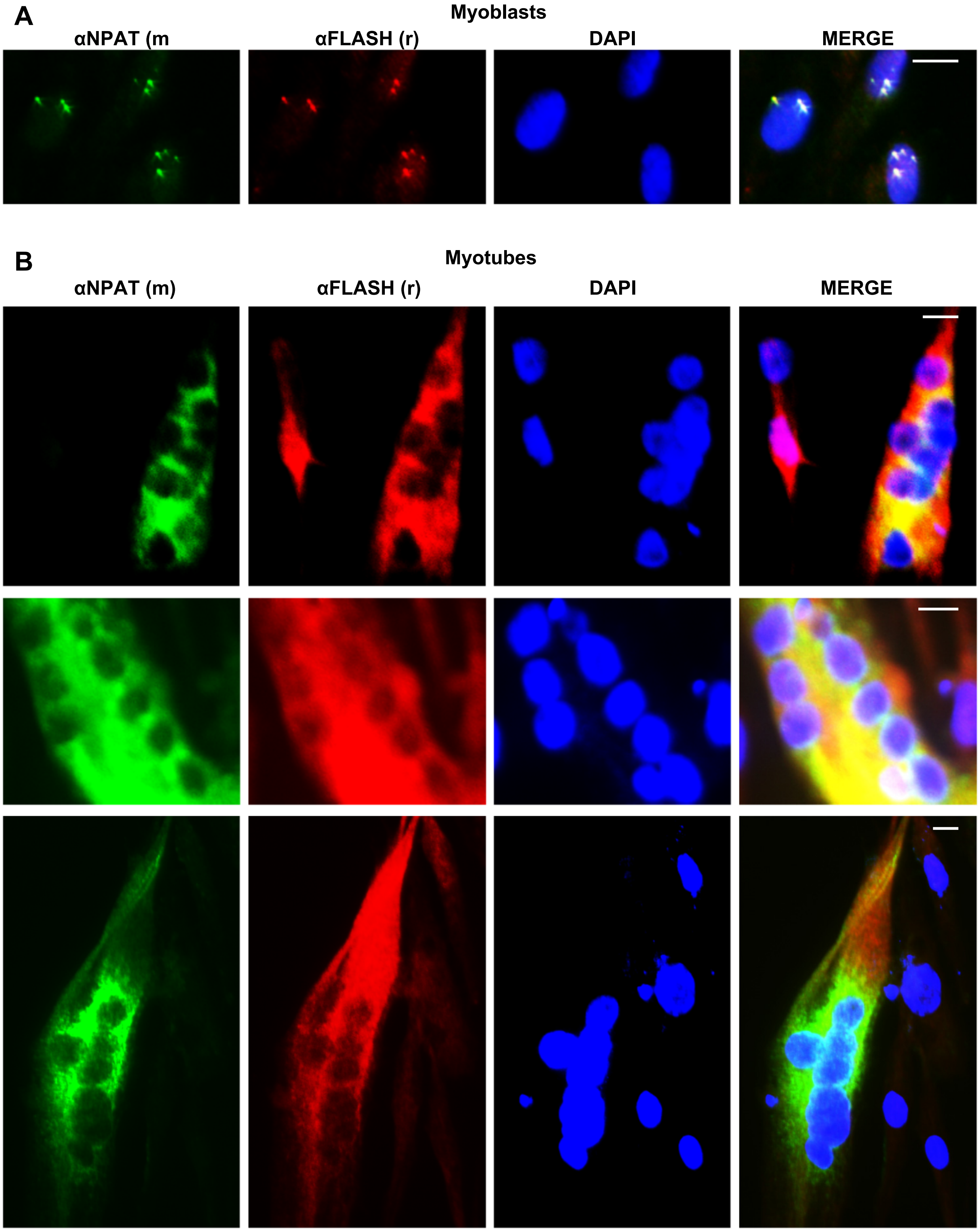

Fig. S6. A and B. Intracellular localization of NPAT and FLASH in constantly cycling LHCN-M2 myoblasts (panel A) and postmitotic multinuclear LHCN-M2 myotubes (panel B). Scale bars represent 10 μM.

Biology Open • Supplementary information

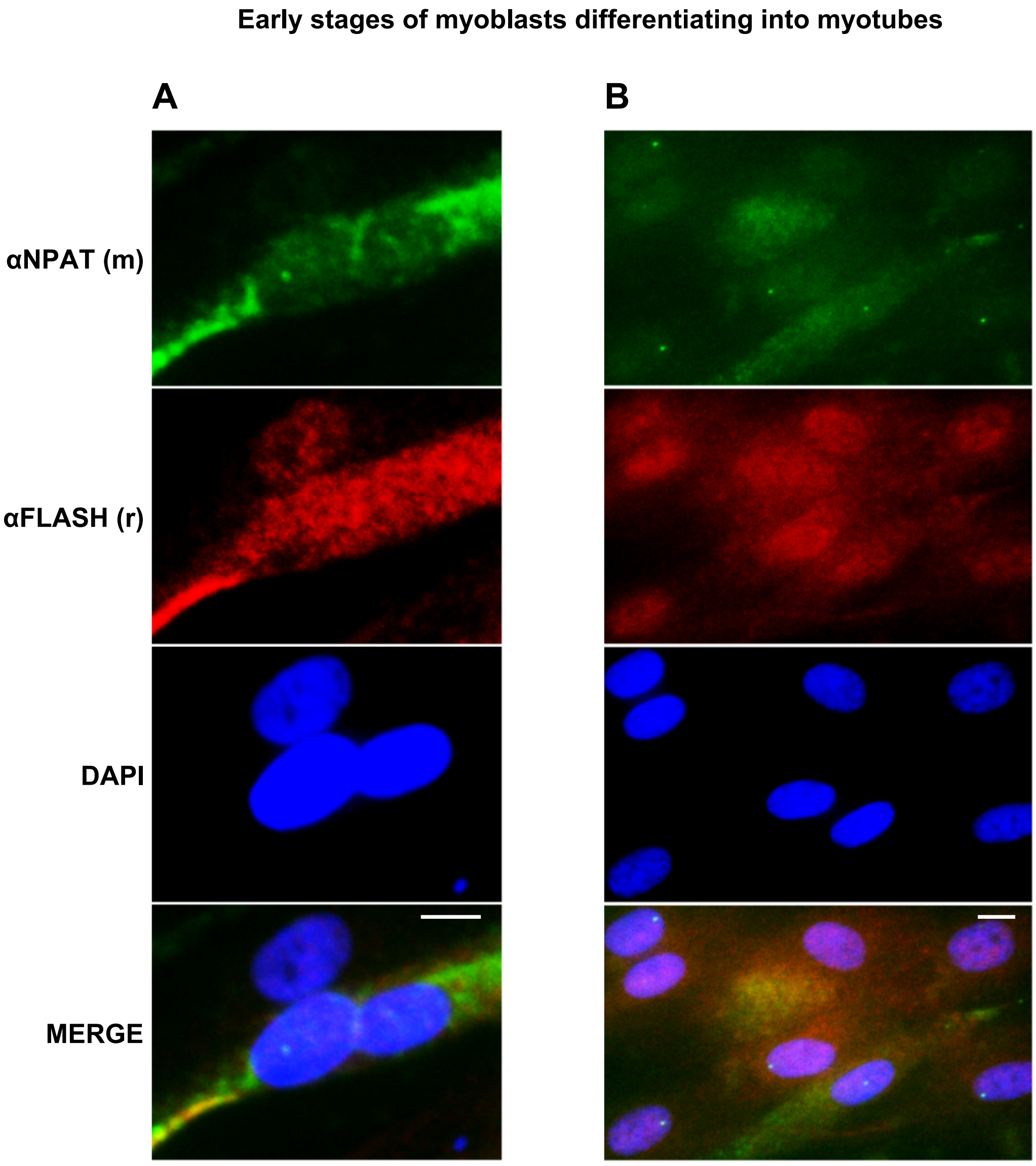

Fig. S7. A and B. Examples of cycling myoblasts differentiating into multinuclear myotubes. These staining patterns likely represent early stages of differentiation and are characterized by the disappearance of HLB and detection of FLASH and NPAT in the nucleoplasm and cytoplasm. Scale bars represent 10 μM.

Biology Open • Supplementary information

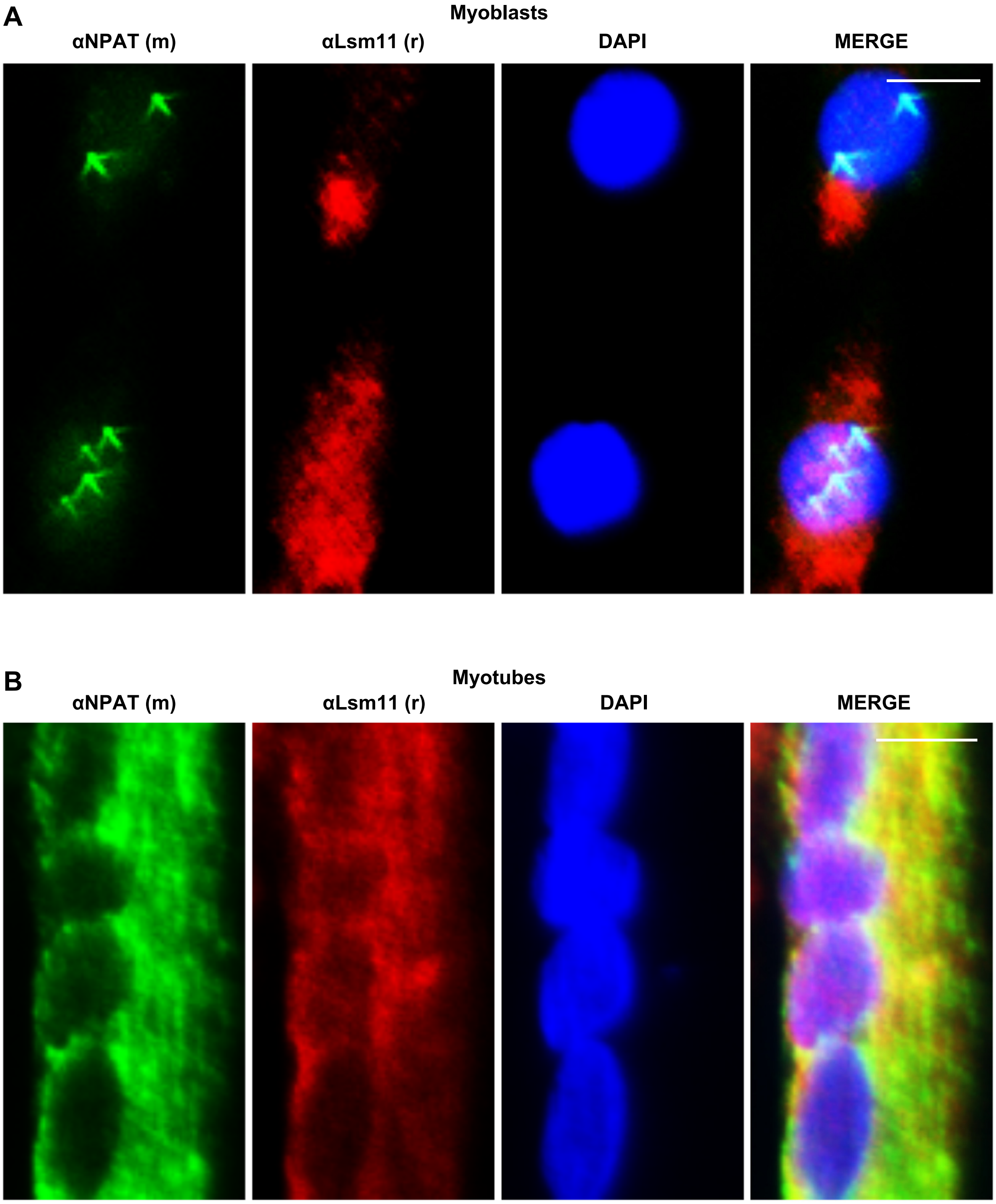

Fig. S8. A and B. Intracellular localization of NPAT and Lsm11 in constantly cycling LHCN-M2 myoblasts (panel A) and postmitotic multinuclear LHCN-M2 myotubes (panel B). Scale bars represent 10 µM.

Biology Open • Supplementary information

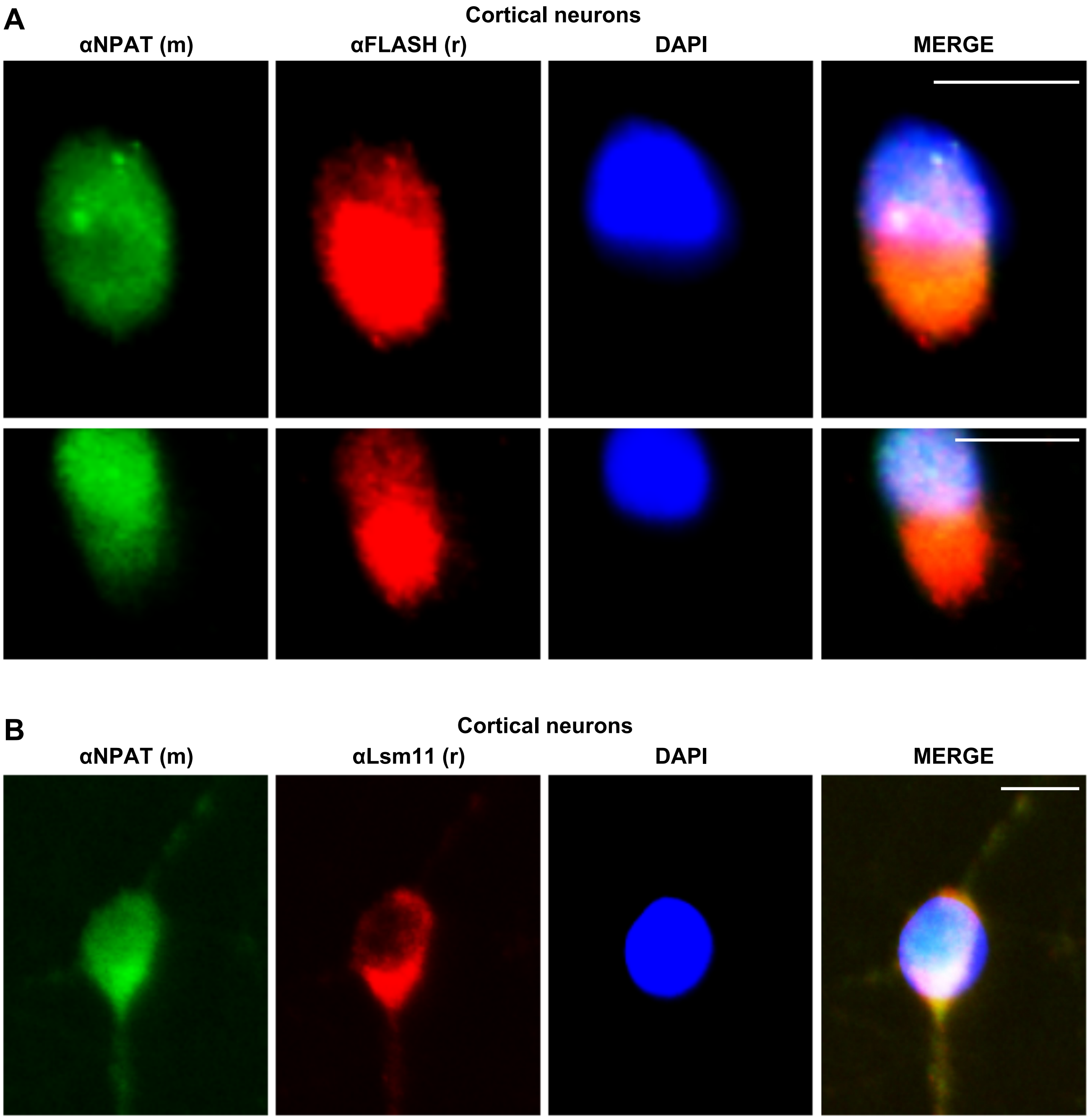

Fig. S9. A and B. Intracellular localization of NPAT and FLASH (panel A) or Lsm11 (panel B) in postmitotic cortical neurons. Scale bars represent 10 µM.

Biology Open • Supplementary information

A

```
1                                                           29
MAADDDNGDGTSLFDVFSASPLKNNDEGSLDIYAGLDSAVSDSASKS
         52 54          62                                  83       89
CVPSRNCLDLYEEILTEEGTAKEATYNDLQVEYGKCQLQMKELMKKF
          101            111                                          139
KEIQTQNFSLINENQSLKKNISALIKTARVEINRKDEEISNLHQR
```

B

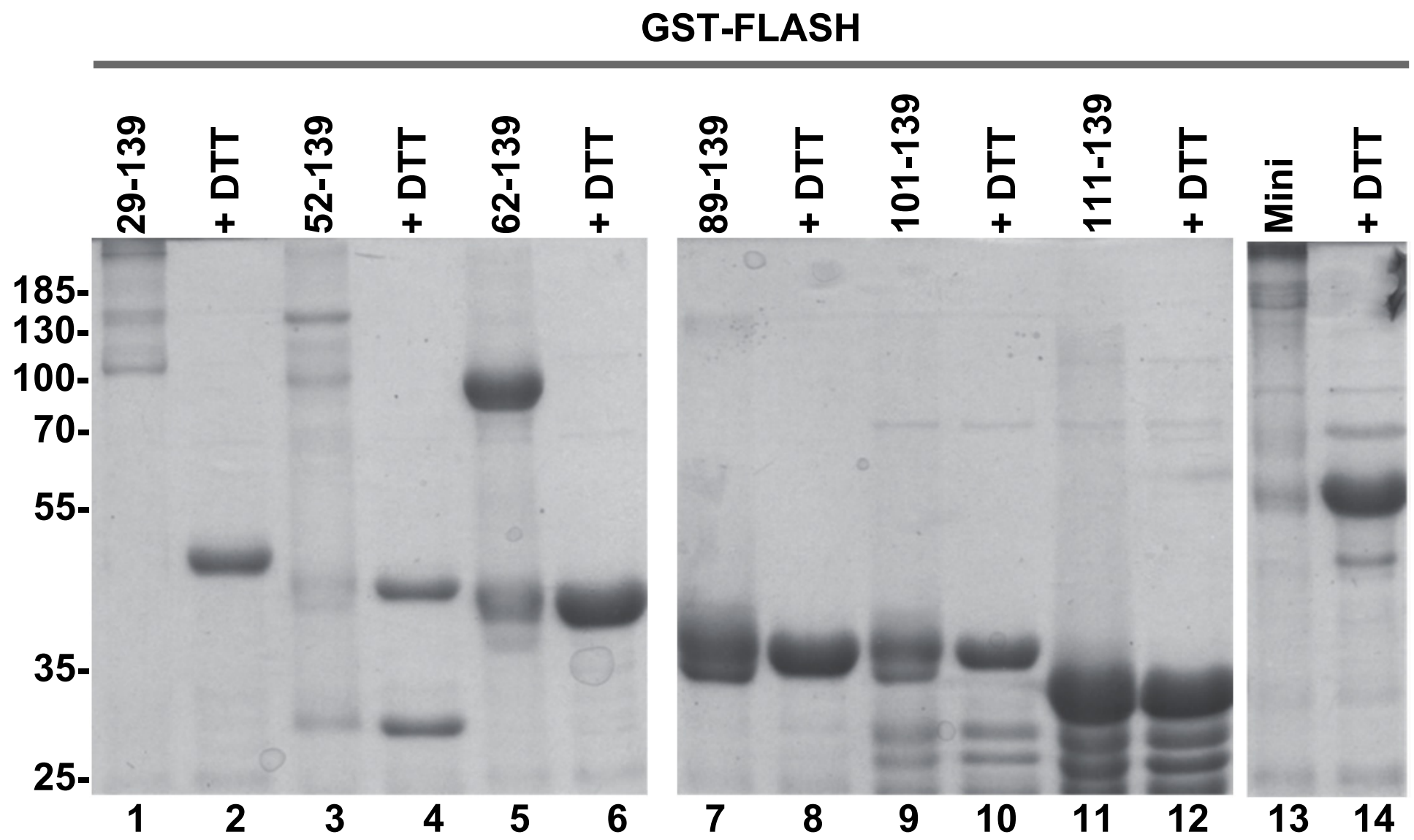

Fig. S10. A. The amino acid sequence of the N-terminal region of FLASH**. B**. The N-terminal region of FLASH forms intermolecular disulfide bonds. The region encompassing amino acids 53-139 folds into a long helix, with residues 71-139 being capable of forming a coiled-coil homodimer {Aik, 2017 #18087}. The helical region contains two cysteines, Cys54 and Cys83 (underlined). In the crystal structure of the homodimer, two Cys83 residues are juxtaposed, raising the possibility that they may spontaneously form a disulfide bridge, although the structure did not provide conclusive evidence for its existence. To test this possibility, we expressed the N-terminal FLASH fragment encompassing amino acids 29-139 and its truncated variants progressively shortened from the N-terminus and compared their ability to multimerize in the absence or presence of a reducing agent. All the proteins were boiled in a sample buffer either containing or lacking DTT, separated in an SDS/polyacrylamide gel and stained with Coomassie Bleu. The two longest FLASH variants tested, encompassing amino acids 29-139 and 52-139 (thus containing two cysteines), in the absence of DTT formed larger complexes migrating near the top of the gel. In the presence of DTT, the complexes were disrupted, suggesting that they were stabilized by a disulfide bond(s). Judging by their molecular size, these complexes likely included dimers, trimers and larger multimers. FLASH variant lacking the first 62 amino acids (62-139 FLASH) formed only a single multimeric form, likely corresponding to a dimer, and its dimerization can be attributed to the sole cysteine in position 83. FLASH 89-139 and FLASH variants with larger N-terminal deletions (FLASH 101-139 and FLASH 111-139) that lack this cysteine were unable to dimerize in the absence of DTT. We also tested the ability to make multimeric forms by mini-FLASH, a natural splice variant in which the entire N-terminal region (AA 1-139) is directly fused with the C-terminal region of 30 residues {Yang, 2014 #17969}. Also, this protein when loaded onto the gel in the absence of DTT migrated as group of larger multimeric complexes, likely stabilized by disulfide bridges involving cysteines 52 and/or 83. Since these covalent bonds are unlikely to form in the reducing environment of the cell, including the nucleus where 3' end processing occurs, our results suggest additional roles for FLASH in the periplasmic space or cel membrane, where formation of disulfide bonds is favored.

Biology Open • Supplementary information

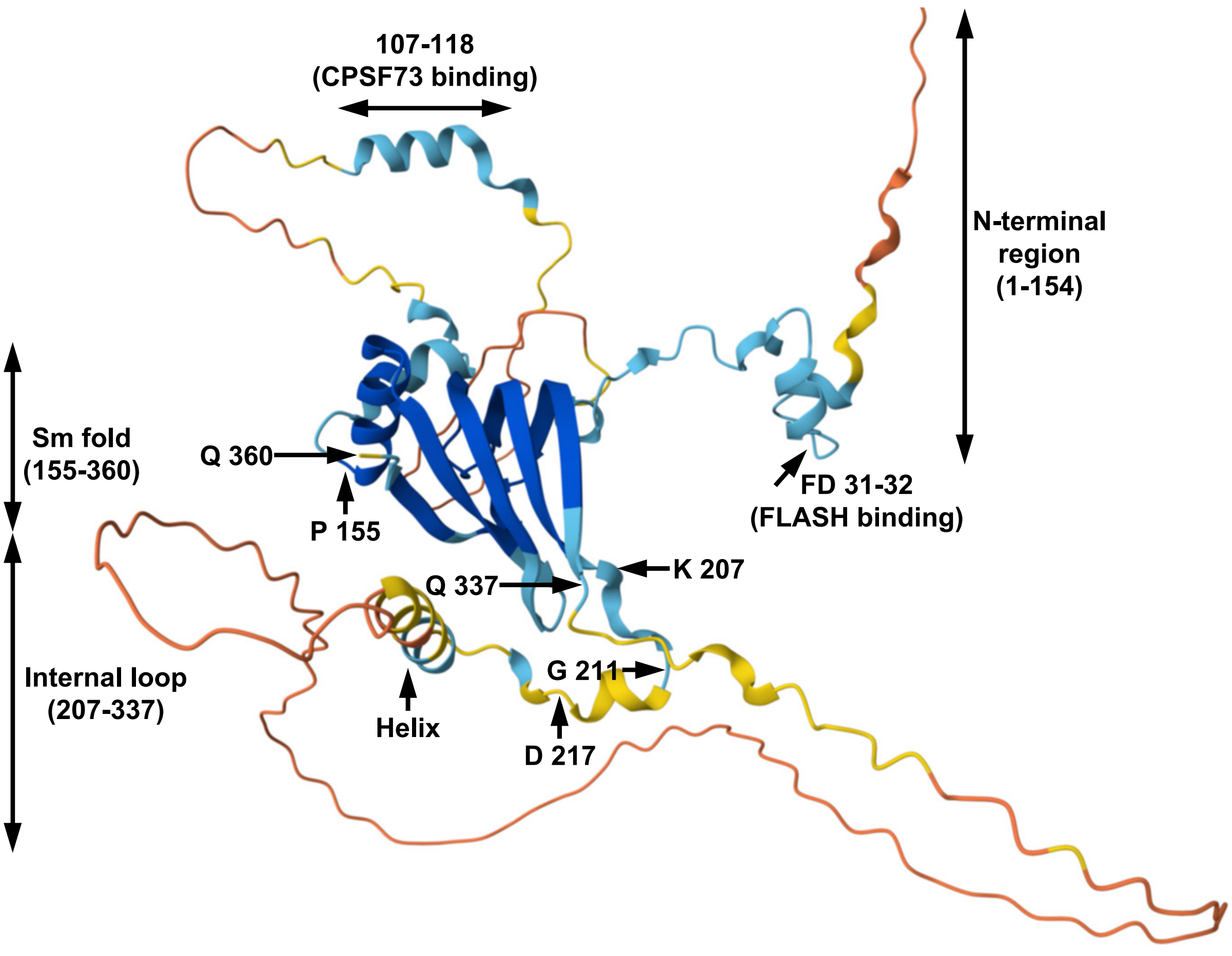

Fig. S11. The structure of human Lsm11, as predicted by AlphaFold 3. The N-terminal region and the Sm fold encompass amino acids 1-154 and 155-360, respectively. The internal loop within the Sm fold encompasses amino acids 207-337. The key amino acids and structural elements are shown.

Biology Open • Supplementary information
